# Immuno- and expression analysis of *Ehrlichia canis* immunoreactive proteins

**DOI:** 10.3389/fvets.2024.1481934

**Published:** 2024-10-21

**Authors:** Jignesh G. Patel, Tian Luo, Xiaofeng Zhang, Jere W. McBride

**Affiliations:** ^1^Department of Pathology, University of Texas Medical Branch, Galveston, TX, United States; ^2^Department of Microbiology and Immunology, University of Texas Medical Branch, Galveston, TX, United States; ^3^Center for Biodefense and Emerging Infectious Diseases, University of Texas Medical Branch, Galveston, TX, United States; ^4^Sealy Institute for Vaccine Sciences, University of Texas Medical Branch, Galveston, TX, United States; ^5^Institute for Human Infections and Immunity, University of Texas Medical Branch, Galveston, TX, United States

**Keywords:** *Ehrlichia*, *E. canis*, immunoreactive protein, Canine monocytic ehrlichiosis (CME), immunodiagnosis, vaccine, tandem repeat proteins (TRPs)

## Abstract

*Ehrlichia canis* is the primary etiologic agent of canine monocytic ehrlichiosis, a serious and sometimes fatal hemorrhagic disease of dogs. Diagnosis of *E. canis* infection is often retrospectively confirmed by serologic detection of antibodies by immunofluorescent microscopy. Our laboratory previously identified numerous major immunoreactive proteins with species-specific linear antibody epitopes that are useful for immunodiagnosis of CME. More recently, we have defined the entire antibody-reactive immunome of *E. canis*, substantially increasing the number of major immunoreactive proteins known to exist. In this study, we analyzed and compared seven recently identified antibody reactive *E. canis* proteins with established diagnostic antigens including tandem repeat proteins TRP19, TRP36 and TRP140 and observed comparable immunoreactivity. Many of these proteins were conserved in different *E. canis* strains. Multiple linear antibody epitopes were mapped in a highly conserved TRP (Ecaj_0126), including within the tandem repeat domain. Temporal antibody responses were examined, and multiple proteins reacted with antibodies in sera as early as 21 days post experimental infection. Host-specific expression of the proteins was examined which revealed that some proteins exhibited higher expression in mammalian cells, while others in tick cells. This study has identified new immunodiagnostic candidates that exhibit different host expression patterns, information which may be useful for developing ultrasensitive immunodiagnostics and effective vaccines for CME.

## Introduction

Canine monocytic ehrlichiosis (CME), primarily caused by *Ehrlichia canis*, is a tick-borne disease in dogs of global importance. *E. canis* is transmitted by the brown dog tick, *Rhipicephalus sanguineus* (1) and CME manifests as a multisystemic disease, which can manifest in multiple forms that include acute, subclinical, or chronic phases (2). The acute phase is characterized by clinical signs and hematologic abnormalities, including depression, anorexia, weight loss, fever, bleeding, thrombocytopenia, and anemia. In the subclinical phase, dogs may spontaneously clear the infection or remain infected and appear clinically healthy for months to years. Finally, some dogs may develop a severe chronic infection characterized by hypoplastic bone marrow, bleeding, and death (3).

Diagnosis of CME can be presumptively determined by visualization of intracytoplasmic *E. canis*-morulae within peripheral blood monocytes; however, this method is the least sensitive and specific (4). Diagnosis is most often confirmed using serologic or molecular methods such as an immunofluorescent antibody assay or PCR (5, 6). Molecular diagnostics such as PCR can be useful, but false negative and positive results are common due to low levels of circulating ehrlichiae in the blood, combined with low PCR sensitivity, contaminants that inhibit PCR, and potential for non-specific amplification (6). Diagnosis of CME by indirect fluorescent-antibody assay (IFA) is considered the serological “gold standard,” but cross-reactive antibodies can make definitive diagnosis by IFA challenging (5). Furthermore, IFA also requires expensive microscopy equipment and subjective interpretation by an experienced microscopist. More recently, molecular immunodiagnosis of *E. canis* infection using peptides containing linear antibody epitopes as diagnostic markers has been utilized in veterinary reference and point of care diagnostic tests (6–8).

In the last two decades, numerous *E. canis* immunoreactive proteins that strongly react with antibodies in sera from infected dogs have been identified and molecularly characterized (9–13). Immunomolecular characterization of these major immunoreactive proteins has revealed several tandem repeat proteins (TRPs) that contain major species-specific linear antibody epitopes located within the tandem repeats (TRs) (10, 12, 14). Three of these TRPs (TRP19, TRP36 and TRP140) have been identified as major immunoreactive proteins that are particularly useful for diagnosis of *E. canis* infection (15, 16). However, genetic variability (i.e., TRP36) can limit the reliability of such antigens depending on the geographic location (17, 18). Thus, the more conserved proteins (i.e., TRP19) are more reliable and preferred for immunodiagnosis of *E. canis* infection (18, 19).

The recently completed *E. canis* immunome has revealed a group of novel immunoreactive proteins (20–22). Most of these proteins are small, secreted effectors with unknown functions. In this study, we evaluated and compared seven recently identified proteins with established TRPs for immunodiagnosis of CME. To understand differences in temporal antibody reactivity and diagnostic sensitivity, we also examined the reactivity with experimentally infected dog sera and determined expression levels of these immunoreactive proteins in *E. canis*-infected tick (ISE6) and mammalian (DH82) cells.

## Materials and methods

### Gene synthesis and cell-free expression

*Ehrlichia canis* (Jake Strain) gene sequences used in the study are available in the Integrated Microbial Genomes (IMG).^1^ *E. canis* gene synthesis was performed (GenScript, Piscataway, NJ, USA) and the genes were cloned into a pIVEX-2.3d or pET-14b vector containing a T7 promoter/terminator and a 6× His-tag sequence. Plasmids with cloned genes were lyophilized and stored at −20°C prior to use.

Cell-free expression of the *E. canis* immunoreactive proteins was performed using the S30 T7 High-Yield Protein Expression System (Promega, Madison, WI, USA). Plasmids were transformed into Stellar competent cells (Takara, Mountain View, CA, USA) and plasmid was extracted and purified using QIAprep Spin Miniprep Kit (Qiagen, Germantown, MD, USA) as previously described (21). The recombinant plasmid was mixed with *E. coli* extract, a premix and reaction mixture and incubated at 37°C with agitation at 750 rpm for 3 h. The cell-free expressed protein was stored at 20°C until use.

### Dog sera

A panel of 15 naturally infected dog sera gifted from United States, Colombia, and Brazil that were confirmed positive for *E. canis* antibody by IFA were used in this study. Sera collected from a dog experimentally infected with *E. canis* (needle inoculation) on days 0, 7, 14, 21, 28, 35, 42 and 56 post infection was used to assess temporal antibody responses to the proteins (16).

### ELISA

Immunoreactivity and diagnostic sensitivity of cell-free expressed *E. canis* proteins were evaluated by ELISA as described previously (21, 22). Briefly, anti-His-antibody coated ELISA plates (GenScript) were blocked with Starting Block Blocking Buffer (Thermo Fisher) at room temperature for 20 min with agitation (300 rpm). After washing twice with PBS-Tween 20 (0.05%) (PBST), plates were coated with cell-free expression lysate containing His-tagged recombinant proteins (50 μL) diluted (1:50) in blocking buffer (TBST, 2% nonfat dry milk) and incubated overnight at 4°C. The plates were washed five times and diluted dog sera (1:200, 50 μL) were added to each well and incubated at room temperature for 1 h with agitation. Plates were washed five times and incubated with alkaline phosphatase-labeled goat anti-dog IgG (H + L) secondary antibody (100 μL; 1:5000, KPL, Gaithersburg, MD), and incubated for 1 h at room temperature with agitation. The plates were washed and BluePhos substrate (100 μL; KPL) was added and incubated for 30 min in the dark with agitation. Color development was measured at *A*_650_ on a VersaMax microplate reader (Molecular Devices) and data analyzed by Softmax Pro 7 software (Molecular Devices). The final optical density (OD_650_) was determined after subtracting OD_650_ value of the negative control (cell-free lysate from empty vector). Positive and negative dog sera were included as controls.

### Epitope mapping

Linear epitopes in the *E. canis* TRP (Ecaj_0126) were mapped with overlapping 25 amino acid peptides (GenScript, Piscataway, NJ, USA) representing the entire open reading frame, except three 35 amino acid TRs which were synthesized separately. Lyophilized peptides were resuspended in molecular grade water (1 mg/mL) and ELISA plates (MaxiSorp; NUNC, Roskilde, Denmark) were coated (1 μg/mL) overnight at 4°C. Plates were washed three times with TBST and blocked with 10% horse serum in TBST for 1 h at room temperature with agitation. The ELISA was performed as previously described (14).

### Cell culture

*Ehrlichia canis* (Jake strain) was propagated in DH82 cells (canine macrophage-like cells) with minimal essential medium (Gibco, Grand Island, NY, USA) supplemented with 10% fetal bovine serum (HyClone, Logan, UT, USA), 1% HEPES (Sigma Chemical Co., St. Louis, MO, USA), 1% sodium pyruvate (Sigma), and 1% nonessential amino acids (Sigma) at 37°C in a humidified 5% CO_2_ atmosphere. ISE6,which is a tick *Ixodes scapularis* embryo-derived cell line, was obtained from Dr. Ulrike Munderloh (University of Minnesota) (23) and maintained in L15B300 medium supplemented with 10% fetal bovine serum (GeminiBio, Sacramento, CA, USA), 10% tryptose phosphate broth (BD, Sparks, MD, USA) and 1% bovine lipoprotein cholesterol concentrate (MP Biomedicals, Irvine, CA, USA) at 34°C as previously described (24). ISE6 cells were infected [multiplicity of infection (MOI) = 10] with host cell-free *E. canis* derived from infected DH82 cells.

### *Ehrlichia canis* antigen

*Ehrlichia canis* antigen for Western blot analysis was prepared as described previously (17). Briefly, infected cells (DH82 and ISE6) were collected when morulae were observed in all cells after Diff-Quik staining. Cells were then centrifuged at (5,000 × *g* for 15 min) and resuspended in phosphate-buffered saline (PBS). Cells were ruptured by sonication, twice (40 Hz) for 10 s, and large cell debris was pelleted by centrifugation (1,500 × *g* for 10 min) at 4°C. The supernatant was centrifuged (10,000 × *g* for 15 min) at 4°C to collect cell-free ehrlichiae. The pellet was washed in PBS, centrifuged (10,000 × *g* for 15 min) at 4°C, and resuspended in PBS. BCA protein assay (Pierce Biotechnology, Rockford, IL, USA) was performed to determine the protein concentration. Cell lysate prepared from the uninfected cells was used as a negative control.

### Antibodies

Polyclonal rabbit antibodies were commercially generated by immunizing rabbits with peptides derived from respective *E. canis* immunoreactive proteins (GenScript). The antisera were used for Western blot analysis and immunofluorescence microscopy.

### Gel electrophoresis and Western blotting

Purified *E. canis* antigen was solubilized in LDS sample buffer containing the reducing agent dithiothreitol (Invitrogen), heated at 70°C for 10 min, and separated by sodium dodecyl-sulfate polyacrylamide gel electrophoresis (SDS-PAGE) in 3-N-morpholinopropanesulfonic acid (MOPS) running buffer under reducing conditions with 4 to 20% gradient Bis-Tris acrylamide gels (NuPAGE; Invitrogen). Proteins were transferred to a nitrocellulose membrane (Protran BA85, 0.45-μm pore size; Whatman, Florham Park, NJ, USA) using a semidry transfer apparatus (Bio-Rad, Hercules, CA, USA). Membranes were blocked for 1 h with TBST blocking buffer (5% nonfat milk). *E. canis* antisera were diluted (1:100) in the blocking buffer and incubated for 1 h at room temperature with shaking. Membranes were washed 3 times with TBST, 5 min each and an affinity-purified alkaline phosphatase-labeled goat anti-rabbit IgG (H & L) (KPL) secondary antibody (1:5000) in blocking buffer was applied and incubated for 1 h. After washing, 5-bromo-4-chloro-3-indolyl-phosphate and nitroblue tetrazolium (BCIP-NBT) substrate (KPL) was applied to visualize bound antibody. Densitometry was performed for Western blot bands of each protein using ImageJ software and the fold-changes of *E. canis* proteins were determined relative to cell actin.

### Immunofluorescence microscopy

Antigen slides were prepared from DH82 or ISE6 cells infected with *E. canis*. Infected cells were applied to 12-well Teflon coated slides, air dried and fixed with 4% paraformaldehyde (PFA) for 20 min at room temperature and washed twice in PBS. Cells were permeabilized with 0.2% Triton X-100 in PBS (BSA) for 15 min and washed. Image-It Signal Enhancer (Invitrogen) was applied to the cells for 30 min, followed by BlockAid (Invitrogen) for 30 min. Cells were then incubated with antigen-specific antisera (1:100) in blocking buffer for 1.5 h, washed three times with PBST, and incubated with goat anti-rabbit IgG (H + L) Alexa Fluorophore Plus 488 secondary antibody (1:200) for 30 min in the dark. The slides were washed thrice and mounted with ProLong Glass Antifade Reagent (Invitrogen). Immunofluorescence images were captured with an Olympus BX61 epifluorescence microscope and analyzed using Slidebook software (v.5.0; Intelligent Imaging Innovations, Denver, CO, USA).

### Real time quantitative PCR (qPCR)

DH82 and ISE6 cells were cultured in T-25 flasks (Cellstar) to 90–95% confluency and incubated with cell-free *E. canis* at a multiplicity of infection (MOI) of 10. Samples were collected daily for DH82 (5 days) and ISE6 cells (8 days), and the absolute *E. canis dsb* copy number was determined by real-time qPCR and plotted against the standard curve, as previously described (25). Briefly, cells were washed with PBS and lysed in SideStep Lysis and Stabilization Buffer (Agilent Technologies, Santa Clara, CA, USA), and real-time quantitative PCR (qPCR) amplification was performed using Brilliant II SYBR Green Mastermix (Agilent), forward primer (5-GCTGCTCCACCAATAAATGTATCCCT-3), and reverse primer (5-GTTTCATTAGCCAAGAATTCCGACACT-3), using a CFX96 Touch Real Time PCR System (BioRad).

## Results

### Comparison of protein immunoreactivity and sensitivity

**Figure 1:**
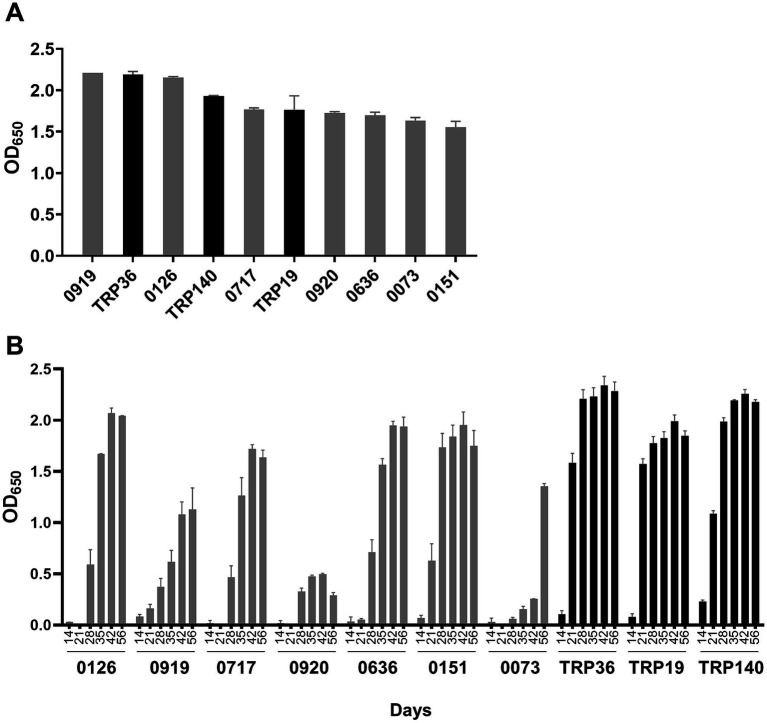
Comparison of *E. canis* major immunoreactive proteins by ELISA. **(A)** *E. canis* protein immunoreactivity. Cell-free expressed immunoreactive proteins were probed with pooled sera from 15 dogs naturally infected with *E. canis*. **(B)** Temporal antibody responses to *E. canis* immunoreactive proteins. Cell-free expressed proteins were probed with sera from a dog (#34) experimentally infected (needle inoculation) with *E. canis* collected at indicated intervals. These proteins did not react with serum from the uninfected dog (#34) as a negative control. ELISA OD values represent the mean optical density reading from triplicate wells (±SD) after background subtraction (IVTT reaction with empty plasmid template).

Immunodominant *E. canis* proteins (*n* = 18) were recently identified by our laboratory in addition to those (i.e., TRPs, Anks, OMP-1) that have been previously reported (9, 11–13). In this study, we selected seven proteins that exhibited the strongest immunoreactivity for further evaluation and compared them to well defined immunoreactive proteins including, TRP19, TRP36 and TRP140. Consistent with our earlier findings, TRP19, TRP36 and TRP140 reacted with 15 naturally infected CME dog sera. By ELISA, TRP36 and two newly identified proteins (Ecaj_0919 and Ecaj_0126) reacted strongly with the dog sera (mean OD_650_ > 2.0), and 5 others (Ecaj_0717, Ecaj_0920, Ecaj_0636, Ecaj_0073 and Ecaj_0151) were slightly lower and similar to TRP140 and TRP19 (mean OD_650_ > 1.7). The sera from naturally infected dogs did not react with cell-free expressed negative control protein (OD_650_ < 0.1) and the cell-free expressed *E. canis* proteins (including TRPs) did not react with sera from uninfected dog. Immunoreactivity of these proteins was determined by ELISA and ranked according to mean OD_650_ (Figure 1A).

To understand the potential differences in diagnostic sensitivity for detection of antibodies generated early in infection, the *E. canis* proteins were examined using convalescent *E. canis* dog sera collected from an experimentally infected dog on days 0, 7, 14, 21, 28, 35, 42, and 56. Dog sera obtained prior to infection (day 0) was used as a negative control. As previously reported (15), TRP36 and TRP140 were most sensitive as both proteins reacted with antibodies in dog sera as early as day 14 (Figure 1B). TRP19 and Ecaj_0919 and Ecaj_0151 reacted with antibodies on day 21. All other immunodominant proteins reacted with antibodies at day 28 except Ecaj_0073, which reacted with antibody at day 35.

### Genetic diversity of *Ehrlichia canis* proteins

We investigated the genetic diversity of the *E. canis.* Proteins (including TRPs) using a BLAST sequence analysis. Protein sequences derived from *E. canis* Jake strain (USA) and *E. canis* YZ-1 (China), and orthologous sequences from *E. minasensis* are shown in Table 1. *E. minasensis* is a relatively new *Ehrlichia* species that is the closest relative to *E. canis* (26). Four of the 7 newly identified proteins in *E. canis* Jake strain were identical to the homologs in the *E. canis* YZ-1 strain, while three proteins had minor differences in the amino acid sequence length and percent identity. TRP19 was very conserved and TRP36 and TRP140 were less conserved as previously reported (18). Compared to orthologs in *E. minasensis*, the *E. canis* TRPs varied significantly in amino acid identity (53–70%). However, *E. minasensis* orthologs of two newly discovered proteins (Ecaj_0073 and Ecaj_0151) were identical in length and had 88 and 94% sequence identity, respectively with *E. canis*. Orthologs of five remaining newly identified *E. canis* proteins had variations in sequence length and their amino acid identity was lower (ranging 37–80%) between strains. These data indicate that many of the recently discovered antibody reactive proteins are highly conserved among *E. canis* strains, but diverse in the closest *E. canis* relative, *E. minasensis.*

**Table 1:** Amino acids and percent identify of *Ehrlichia canis* immunoreactive proteins.

*E. canis* (Jake) proteins (No. of amino acids)	*E. canis* (*YZ-1*) Amino acids (% identity to Jake)	*E. minasensis* Amino acids (% identity to Jake)
Ecaj_0126 (671 AA)	671 (100)	740 (77)
Ecaj_0919 (120 AA)	117 (87)	119 (60)
Ecaj_0717 (226 AA)	227 (85)	223 (37.84)
Ecaj_0920 (182 AA)	168 (93)	88 (91)
Ecaj_0636 (98 AA)	98 (100)	104 (80)
Ecaj_0073 (92 AA)	92 (100)	92 (88)
Ecaj_0151 (205 AA)	205 (100)	205 (94)
Ecaj_0113 [TRP19] (137 AA)	137 (98)	139 (53)
Ecaj_0017 [TRP140] (688 AA)	616 (84)	513 (70)
Ecaj_0109 [TRP36] (207 AA)	264 (82)	229 (61)

### Tandem repeat protein (Ecaj_0126) linear epitope mapping

**Figure 2:**
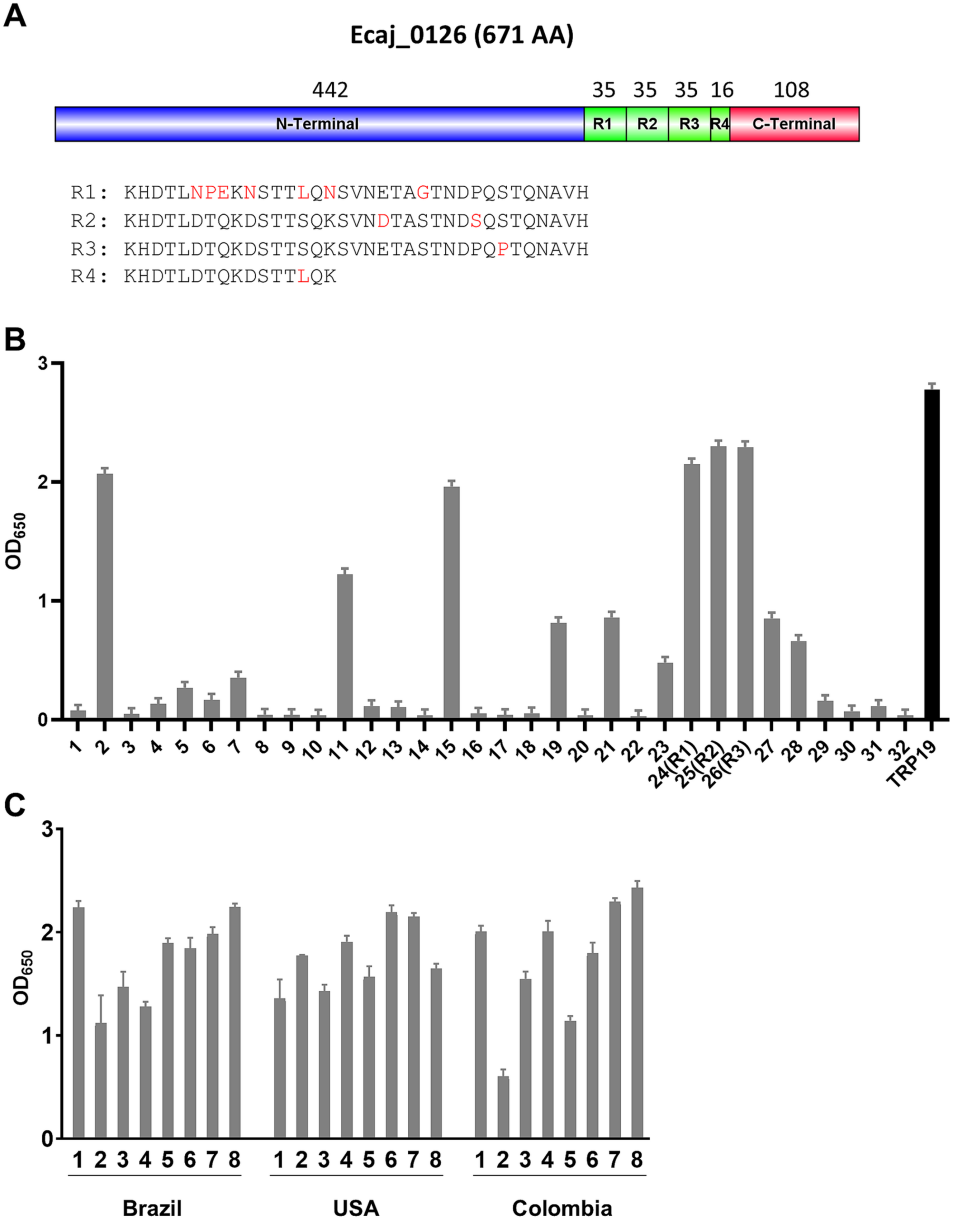
Linear epitope mapping of Ecaj_0126. **(A)** Schematic of the *E. canis* TRP (Ecaj_1026) showing domains and location of TRs. Number of amino acids is shown above each domain. Alignment of amino acid sequences of four TRs is shown below and heterogenous residues are showed in red. R = repeat. **(B)** Immunoreactivity of overlapping synthetic peptides as determined by ELISA with pooled CME dog sera from USA, Colombia and Brazil. TRP19 peptide was used as a positive control. ELISA OD values represent the mean optical density from triplicate wells (±SD) after background subtraction. **(C)** Immunoreactivity of Ecaj_0126 R2 peptide with 24 CME dog sera from USA, Columbia and Brazil.

Our recent study defining the *E. canis* immunome revealed a highly immunoreactive *E. canis* protein (Ecaj_0126) (22). In this investigation, we determined that Ecaj_0126 contained a C-terminal TR domain (Figure 2A). Ecaj_0126 has a predicted mass of ~70 KDa (671 amino acids) and contains three nearly identical 35 amino acid repeats and a fourth partial repeat (16 amino acids). To identify linear antibody epitopes in Ecaj_0126, we screened overlapping peptides (25 amino acids; 6 amino acid overlap) and the 3 full length repeats (35 amino acids) from the TR region (Table 2). Multiple strongly immunoreactive peptides were identified with Ecaj_0126 indicating the presence of linear antibody epitopes (Figure 2B). Further investigations of these immunoreactive epitopes with additional CME dog sera (*n* = 10) consistently found strong antibody reactivity with the TR peptides. We then screened the Ecaj_0126 R2 peptide with 8 CME dog sera collected from different North and South American countries (USA, Colombia, and Brazil). We observed consistent antibody reactivity with all dog sera, indicating that the TR epitope is conserved among geographically dispersed *E. canis* strains (Figure 2C).

**Table 2:** Ecaj_0126 peptides for epitope mapping.

Peptide	Amino acid sequence
1	MTSNTSIPKNHEYSFKLDIGENLYF
2	GENLYFFCNHNVHKVKIITEDNTEI
3	EDNTEITMPSKNYFFVGDKFYAPYN
4	FYAPYNNYFYDNYLNIPAEYRYIKV
5	YRYIKVDHMQYRTTNNEQPQDFYNL
6	QDFYNLVLCDKNGEEYRYNYYKFYI
7	YYKFYIKPENIIEKSAEINLKEYYN
8	LKEYYNIQQLKEGAPLFKIVSEQPN
9	VSEQPNNTTKASTALILDISSNQKF
10	SSNQKFAKLSPEALQYKHYLDRNSP
11	LDRNSPTYDTFTLSYSDIRKHHVDE
12	KHHVDEQEKINLHNIRDDILQAEME
13	LQAEMENNPIFLVIQDGKYFFTDVK
14	FFTDVKQDQPLTTSYNTALKVLASA
15	KVLASANFQINNVPNDNCYVDMHKK
16	VDMHKKFIFKITKSNLHTEHDNSKN
17	HDNSKNLASITLEGKEIPLISNDDD
18	ISNDDDTQIFYDDFSFKCYQNFTQV
19	QNFTQVFNYDEPIIGLDKDFYEPIK
20	FYEPIKEKLSSNNIYITIKSDEQNH
21	SDEQNHIKTYFSDKQGNHILDLPNT
22	LDLPNTKLTEYLSTMLPLGDFSNEV
23	DFSNEVLNTHIEDIAHQKLSDTTQ
24 (R1)	KHDTLNPEKNSTTLQNSVNETAGTNDPQSTQNAVH
25 (R2)	KHDTLDTQKDSTTSQKSVNDTASTNDSQSTQNAVH
26 (R3)	KHDTLDTQKDSTTSQKSVNETASTNDPQPTQNAVH
27	LVSEEHNINKSNTNINVEQNIVYFP
28	NIVYFPLSREHVSIVDNIEQNKHHV
29	QNKHHVSFNLTYEEMLNFYEAVKEQ
30	EAVKEQYSYDEVLIAYNNIFKNYGR
31	FKNYGREQKNDNIYIDGDNHIFIEN
32	HIFIENHDFGILQ

### Temporal expression of *Ehrlichia canis* proteins in mammalian and tick cells

**Figure 3:**
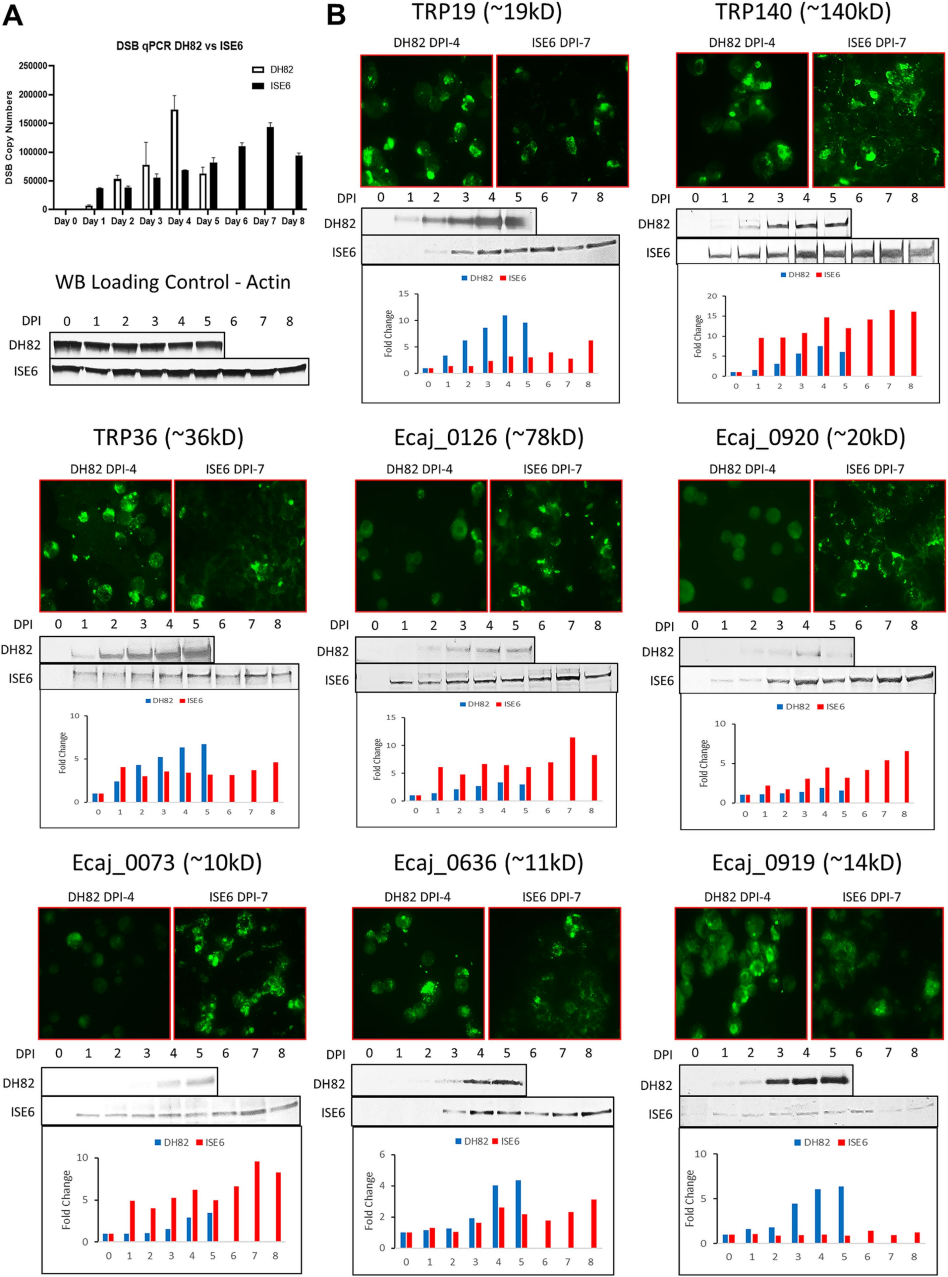
Host-specific and temporal expression of *E. canis* immunoreactive proteins. **(A)** Real time qPCR quantification of *E. canis* temporal growth in DH82 and ISE6 cells. **(B)** Immunofluorescent microscopy and Western immunoblot analysis of *E. canis* immunoreactive protein expression in DH82 and ISE6 cells. Immunofluorescent micrographs correspond to day 4 (DH82) and day 7 (ISE6) of infection. Antibodies used in the analysis were produced in rabbits against protein-specific peptides. Densitometry was performed for Western blot bands of each *E. canis* protein and cell actin using ImageJ software and the chart bars show the fold-changes of each protein relative to actin.

By real time qPCR, *E. canis* replication increased more rapidly in DH82 cells, peaking at day 4 compared to ISE6 cells, which exhibited a slower (peak at day 7) more linear growth curve (Figure 3A). To examine differences in the protein expression levels of the *E. canis* TRPs and newly identified immunoreactive proteins, IFA and Western blot analyses were performed using *E. canis-*infected DH82 cells and ISE6 cells (Figure 3B). After determining the growth curve in both cell lines, we prepared *E. canis* antigen slides using DH82 cells (day 4) and ISE6 cells (day 7). By immunoblot, four proteins (TRP140, Ecaj_0126, 0920 and 0073) exhibited higher overall expression levels in ISE6 cells compared to DH82. In contrast, TRP19, TRP36, Ecaj_0636 and 0919 exhibited higher overall expression in DH82 cells. IFA images were consistent with Western immunoblot densitometry on days 4 (DH82) and 7 (ISE6) post infection.

## Discussion

Defining the antibody-reactive antigens of *Ehrlichia* spp. and their molecular characteristics is important for developing the most sensitive and specific immunodiagnostics and perhaps more importantly, protective vaccines. Defining *Ehrlichia* immunoreactive proteins has progressed over the last 25 years culminating in the full characterization of the antibody-reactive immunomes of *E. chaffeensis* and *E. canis* (20–22). A total of 18 new proteins were identified in *E. canis* that were previously unknown (22). Prior to completing the immunome, a small subset of *E. canis* proteins that react strongly with antibodies in sera from infected dogs were defined and considered major immunoreactive proteins, including three *E. canis* TRPs (TRP140, TRP36 and TRP19) and corresponding orthologs in *E. chaffeensis* (TRP120, TRP47 and TRP32). A more detailed immunomolecular analysis revealed major linear species-specific epitopes within the TR units of these proteins. Notably, peptides representing these epitopes are sensitive and specific for detection of antibodies, some of which are currently used as diagnostic markers in veterinary reference and point of care tests (7, 8).

*E. canis* is a globally distributed disease and many studies have identified genetic variation in major immunoreactive proteins such as TRP36 (18, 19). Conversely, TRP19 appears to be relatively conserved which is beneficial for developing diagnostic tests that are reliable worldwide (19). Notably, many of the new major immunoreactive proteins (Ecaj_0126, 0636, 0073, 0151) examined in this study appear to be highly conserved, whereas others exhibit some genetic diversity. This suggests that many of these conserved proteins would be particularly useful for reliable diagnosis of CME regardless of geographic location.

We previously reported that TRP36 and TRP19 elicited early antibody responses in dogs experimentally infected with *E. canis* (15, 16). Similarly, we found that several of the new major immunoreactive proteins elicit antibodies 3 weeks post infection. Using sera from an experimentally infected dog, we observed lower antibody responses to some proteins (Ecaj_0073 and Ecaj_0920) than was observed with naturally infected dog sera (Figure 1). The difference in the response we observed with sera from naturally infected dogs may be related to factors associated with tick transmission compared to experimental needle inoculation, the route used to experimentally infect dogs. It is notable that these two proteins exhibited higher expression levels in tick cells compared to mammalian cells in this study which supports this possibility. Nevertheless, all the new major immunoreactive proteins elicit a robust antibody response in naturally infected dogs and suggests that these proteins could be valuable diagnostic and/or vaccine candidates.

Since changes in *Ehrlichia* phenotype in different hosts is not well understood, we examined the expression of these immunoreactive proteins in tick and mammalian host cells. In a previous study we reported *Ehrlichia* gene transcription profiles in tick and mammalian cells and found that *E. chaffeensis* transcriptome expression is higher in tick cells compared to mammalian cells (24). However, TRPs were found to be some of the most highly expressed genes in mammalian cells, which was consistent with the expression data we observed in the current study. Notably, half of the proteins examined in this study demonstrated higher expression levels in tick cells suggesting they may be important for tick infection and transmission. These differences in host expression also illustrate how studies using only ehrlichiae produced in mammalian cells could be biased. Many strongly immunoreactive proteins are more highly expressed in ticks and likely to be important targets for transmission blocking vaccines or elicit earlier antibody responses that would be useful for improving diagnostic sensitivity.

*E. chaffeensis* and *E. canis* TRPs were identified more than a decade ago based on strong antibody reactivity by Western immunoblot (16). Subsequently, immunomolecular analysis and epitope mapping identified species-specific linear antibody epitopes in TRPs that defined the molecular basis of antibody reactivity (12, 14, 27). Notably, TRPs are now known to be secreted effector proteins that have multiple functions and interactions with the host cell including acting as nucleomodulins, ubiquitin ligases and eukaryotic signaling pathway ligand mimics (28). Similarly, a new TRP was identified (Ecaj_0126) that is 671 amino acids in length and has three (35 amino acids) TRs. Like other well-known TRPs, Ecaj_0126 exhibited strong immunoreactivity, and in this study, we identified a linear antibody epitope in the TR of Ecaj_0126. Moreover, we also identified at least 3 major linear epitopes outside the TR domain that exhibited immunoreactivity similar to the TR epitope. Notably, Ecaj_0126 is highly conserved (100%) among dispersed *E. canis* strains, suggesting it could be a very reliable diagnostic antigen regardless of genetic and phenotypic variation among strains. This is further supported by the strong and consistent reactivity of Ecaj_0126 peptides with dog sera from North and South America.

## Data Availability

The original contributions presented in the study are included in the article/Supplementary material, further inquiries can be directed to the corresponding author.

## Footnotes

^1^ http://img.jgi.doe.gov/

## References

[1] Groves MG Dennis GL Amyx HL Huxsoll DL. Transmission of *Ehrlichia canis* to dogs by ticks (*Rhipicephalus sanguineus*). Am J Vet Res. (1975) 36:937–40. PMID: 1147359

[2] Mylonakis ME Harrus S Breitschwerdt EB. An update on the treatment of canine monocytic ehrlichiosis (*Ehrlichia canis*). Vet J. (2019) 246:45–53. doi: 10.1016/j.tvjl.2019.01.015, PMID: 30902188

[3] Mylonakis ME Koutinas AF Breitschwerdt EB Hegarty BC Billinis CD Leontides LS. Chronic canine ehrlichiosis (*Ehrlichia canis*): a retrospective study of 19 natural cases. J Am Anim Hosp Assoc. (2004) 40:174–84. doi: 10.5326/0400174, PMID: 15131097

[4] Mylonakis ME Koutinas AF Billinis C Leontides LS Kontos V Papadopoulos O. Evaluation of cytology in the diagnosis of acute canine monocytic ehrlichiosis (*Ehrlichia canis*): a comparison between five methods. Vet Microbiol. (2003) 91:197–204. doi: 10.1016/S0378-1135(02)00298-5, PMID: 12458168

[5] Waner T Harrus S Jongejan F Bark H Keysary A Cornelissen AW. Significance of serological testing for ehrlichial diseases in dogs with special emphasis on the diagnosis of canine monocytic ehrlichiosis caused by *Ehrlichia canis*. Vet Parasitol. (2001) 95:1–15. doi: 10.1016/S0304-4017(00)00407-6, PMID: 11163693

[6] Harrus S Waner T. Diagnosis of canine monocytotropic ehrlichiosis (*Ehrlichia canis*): an overview. Vet J. (2011) 187:292–6. doi: 10.1016/j.tvjl.2010.02.001, PMID: 20226700

[7] Beall MJ Mainville CA Arguello-Marin A Clark G Lemieux C Saucier J. An improved point-of-care ELISA for the diagnosis of Anaplasmosis and Ehrlichiosis during the acute phase of tick-borne infections in dogs. Top Companion Anim Med. (2022) 51:100735. doi: 10.1016/j.tcam.2022.100735, PMID: 36273749

[8] Qurollo BA Stillman BA Beall MJ Foster P Hegarty BC Breitschwerdt EB. Comparison of *Anaplasma* and *Ehrlichia* species-specific peptide ELISAs with whole organism-based immunofluorescent assays for serologic diagnosis of anaplasmosis and ehrlichiosis in dogs. Am J Vet Res. (2021) 82:71–80. doi: 10.2460/ajvr.82.1.71 33369490

[9] Yu XJ McBride JW Diaz CM Walker DH. Molecular cloning and characterization of the 120-kilodalton protein gene of *Ehrlichia canis* and application of the recombinant 120-kilodalton protein for serodiagnosis of canine ehrlichiosis. J Clin Microbiol. (2000) 38:369–74. doi: 10.1128/JCM.38.1.369-374.2000, PMID: 10618118 PMC88726

[10] Doyle CK Cardenas AM Aguiar DM Labruna MB Ndip LM Yu XJ. Molecular characterization of *E. canis* gp36 and *E. chaffeensis* gp47 tandem repeats among isolates from different geographic locations. Ann N Y Acad Sci. (2005) 1063:433–5. doi: 10.1196/annals.1355.079 16481555

[11] Nethery KA Doyle CK Zhang X McBride JW. *Ehrlichia canis* gp200 contains dominant species-specific antibody epitopes in terminal acidic domains. Infect Immun. (2007) 75:4900–8. doi: 10.1128/IAI.00041-07, PMID: 17682040 PMC2044547

[12] McBride JW Doyle CK Zhang X Cardenas AM Popov VL Nethery KA. Identification of a glycosylated *Ehrlichia canis* 19-kilodalton major immunoreactive protein with a species-specific serine-rich glycopeptide epitope. Infect Immun. (2007) 75:74–82. doi: 10.1128/IAI.01494-06, PMID: 17088359 PMC1828430

[13] McBride JW Yu X Walker DH. Molecular cloning of the gene for a conserved major immunoreactive 28-kilodalton protein of *Ehrlichia canis:* a potential serodiagnostic antigen. Clin Diagn Lab Immunol. (1999) 6:392–9. doi: 10.1128/CDLI.6.3.392-399.1999, PMID: 10225842 PMC103729

[14] Luo T Zhang X McBride JW. Major species-specific antibody epitopes of the *Ehrlichia chaffeensis* p120 and *E. canis* p140 orthologs in surface-exposed tandem repeat regions. Clin Vacc Immunol. (2009) 16:982–90. doi: 10.1128/CVI.00048-09, PMID: 19420187 PMC2708412

[15] Cardenas AM Doyle CK Zhang X Nethery K Corstvet RE Walker DH. Enzyme-linked immunosorbent assay with conserved immunoreactive glycoproteins gp36 and gp19 has enhanced sensitivity and provides species-specific immunodiagnosis of *Ehrlichia canis* infection. Clin Vacc Immunol. (2007) 14:123–8. doi: 10.1128/CVI.00361-06, PMID: 17151186 PMC1797795

[16] McBride JW Corstvet RE Gaunt SD Boudreaux C Guedry T Walker DH. Kinetics of antibody response to *Ehrlichia canis* immunoreactive proteins. Infect Immun. (2003) 71:2516–24. doi: 10.1128/IAI.71.5.2516-2524.2003, PMID: 12704123 PMC153292

[17] Zhang X Luo T Keysary A Baneth G Miyashiro S Strenger C. Genetic and antigenic diversities of major immunoreactive proteins in globally distributed *Ehrlichia canis* strains. Clin Vacc Immunol. (2008) 15:1080–8. doi: 10.1128/CVI.00482-07, PMID: 18480237 PMC2446643

[18] Aguiar DM Zhang X Melo AL Pacheco TA Meneses AM Zanutto MS. Genetic diversity of *Ehrlichia canis* in Brazil. Vet Microbiol. (2013) 164:315–21. doi: 10.1016/j.vetmic.2013.02.015, PMID: 23490559

[19] Nambooppha B Rittipornlertrak A Tattiyapong M Tangtrongsup S Tiwananthagorn S Chung YT. Two different genogroups of *Ehrlichia canis* from dogs in Thailand using immunodominant protein genes. Infection Genet Evol. (2018) 63:116–25. doi: 10.1016/j.meegid.2018.05.027, PMID: 29852293

[20] Luo T Patel JG Zhang X Walker DH McBride JW. *Ehrlichia chaffeensis* and *E. canis* hypothetical protein immunoanalysis reveals small secreted immunodominant proteins and conformation-dependent antibody epitopes. NPJ Vacc. (2020) 5:85. doi: 10.1038/s41541-020-00231-1, PMID: 32963815 PMC7486380

[21] Luo T Patel JG Zhang X Walker DH McBride JW. Immunoreactive protein repertoires of *Ehrlichia chaffeensis* and *E. canis* reveal the dominance of hypothetical proteins and conformation-dependent antibody epitopes. Infect Immun. (2021) 89:e0022421. doi: 10.1128/IAI.00224-21, PMID: 34370510 PMC8519286

[22] Luo T Patel JG Zhang X McBride JW. Antibody reactive immunomes of *Ehrlichia chaffeensis* and *E. canis* are diverse and defined by conformational antigenic determinants. Front Cell Infect Microbiol. (2023) 13:1321291. doi: 10.3389/fcimb.2023.1321291 38264730 PMC10803646

[23] Munderloh UG Liu Y Wang M Chen C Kurtti TJ. Establishment, maintenance and description of cell lines from the tick *Ixodes scapularis*. J Parasitol. (1994) 80:533–43. doi: 10.2307/3283188, PMID: 8064520

[24] Kuriakose JA Miyashiro S Luo T Zhu B McBride JW. *Ehrlichia chaffeensis* transcriptome in mammalian and arthropod hosts reveals differential gene expression and post transcriptional regulation. PLoS One. (2011) 6:e24136. doi: 10.1371/journal.pone.0024136, PMID: 21915290 PMC3167834

[25] Dunphy PS Luo T McBride JW. *Ehrlichia chaffeensis* exploits host SUMOylation pathways to mediate effector-host interactions and promote intracellular survival. Infect Immun. (2014) 82:4154–68. doi: 10.1128/IAI.01984-14, PMID: 25047847 PMC4187855

[26] Cabezas-Cruz A Valdes JJ de la Fuente J. The glycoprotein TRP36 of *Ehrlichia* sp. UFMG-EV and related cattle pathogen *Ehrlichia* sp. UFMT-BV evolved from a highly variable clade of *E. canis* under adaptive diversifying selection. Parasit Vectors. (2014) 7:584. doi: 10.1186/s13071-014-0584-5, PMID: 25499826 PMC4266974

[27] Doyle CK Nethery KA Popov VL McBride JW. Differentially expressed and secreted major immunoreactive protein orthologs of *Ehrlichia canis* and *E. chaffeensis* elicit early antibody responses to epitopes on glycosylated tandem repeats. Infect Immun. (2006) 74:711–20. doi: 10.1128/IAI.74.1.711-720.2006, PMID: 16369028 PMC1346619

[28] Byerly CD Patterson LL McBride JW. *Ehrlichia* TRP effectors: moonlighting, mimicry and infection. Pathogens Dis. (2021) 79:ftab026. doi: 10.1093/femspd/ftab026, PMID: 33974702 PMC8112483

