# Supplementary figures and images for: Immuno- and expression analysis of Ehrlichia canis immunoreactive proteins
Source: Front Vet Sci. 2024 Oct 21;11:1481934. doi: 10.3389/fvets.2024.1481934 (PMC11532101; doi:10.3389/fvets.2024.1481934)

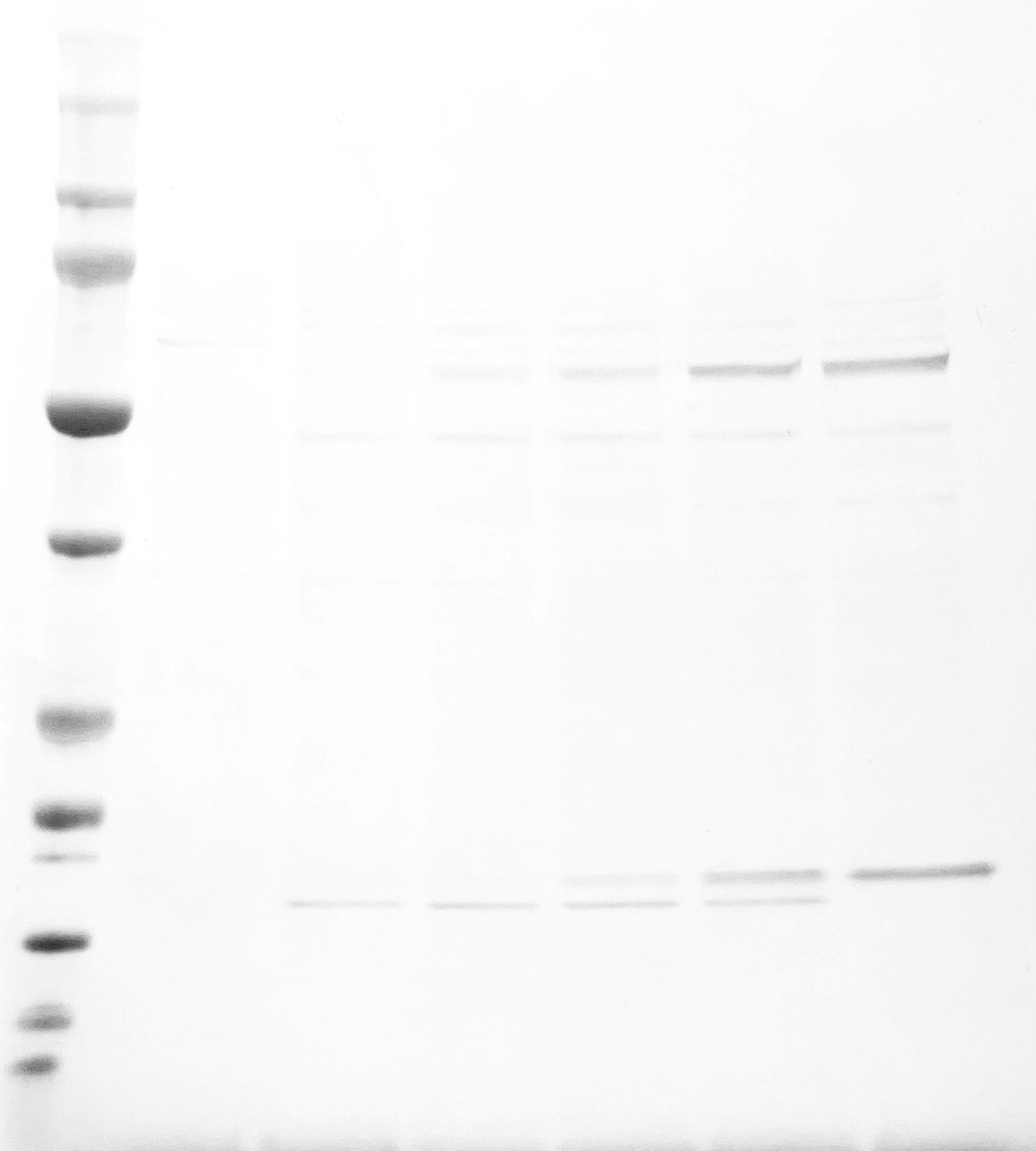

Supplement: Supplementary file 1 [file Data_Sheet_1.zip › supplemental raw WB pictures/DH82 0073 expression 021122.jpg]

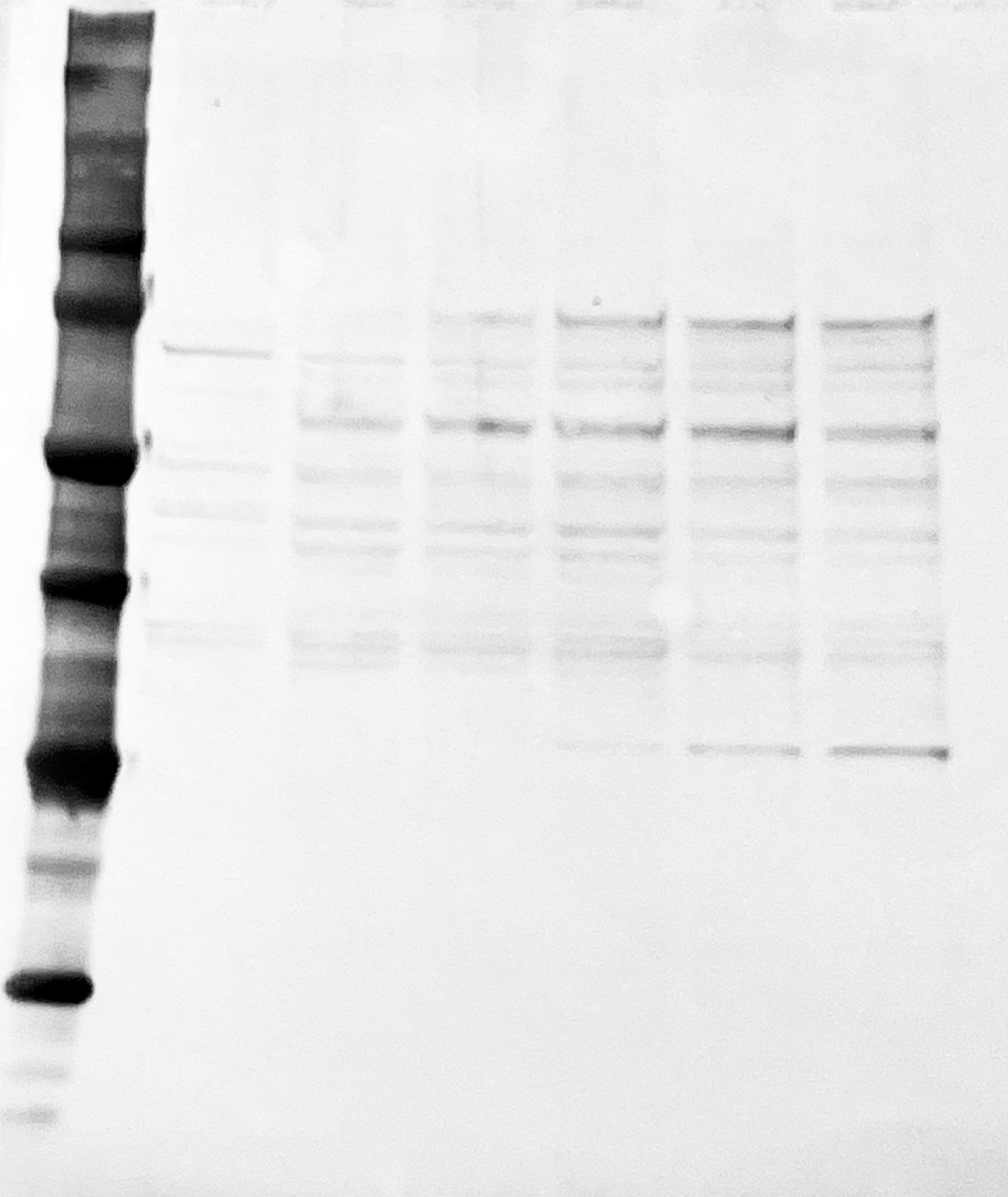

Supplement: Supplementary file 1 [file Data_Sheet_1.zip › supplemental raw WB pictures/DH82 0126 expression 021622.jpg]

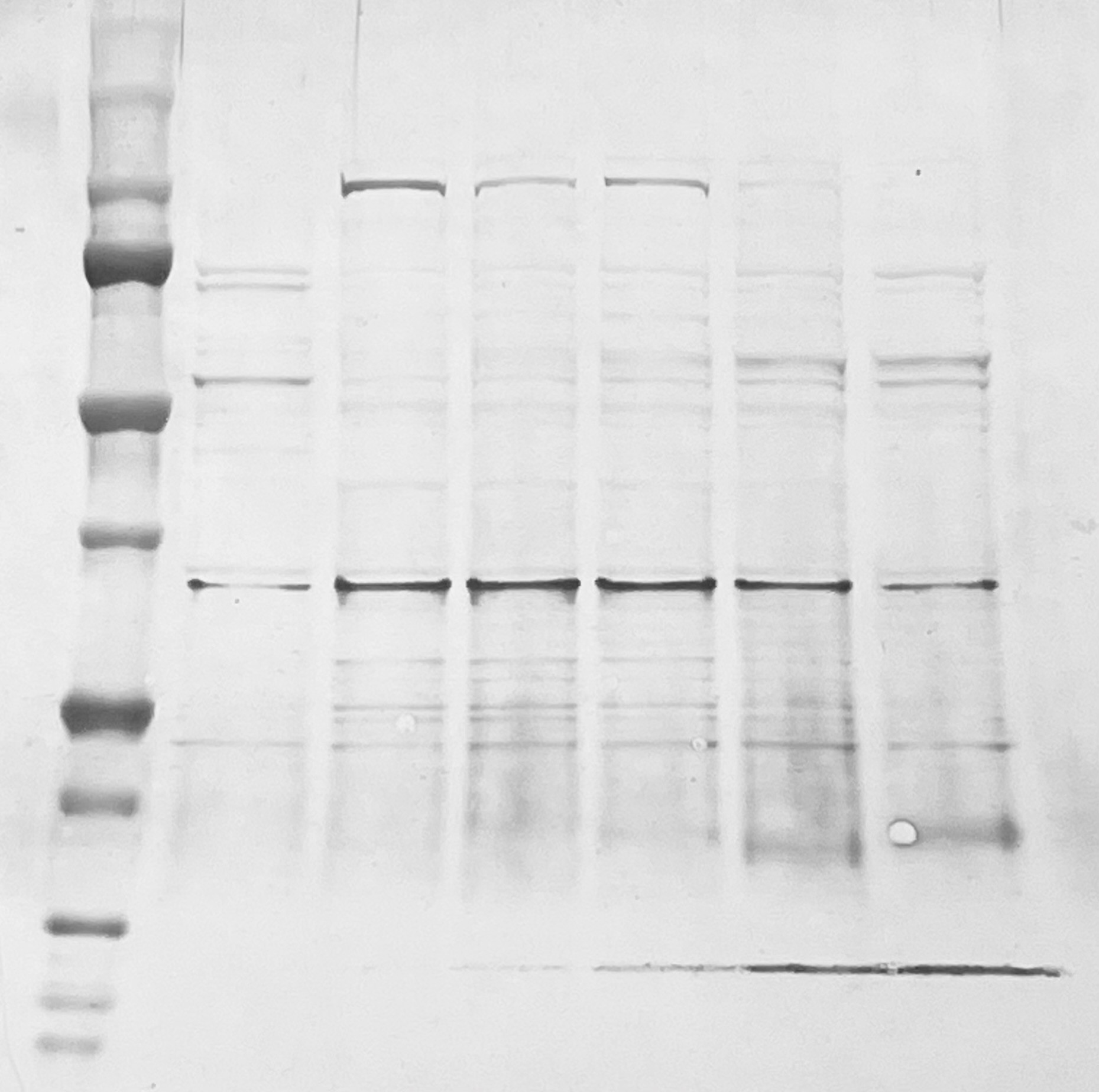

Supplement: Supplementary file 1 [file Data_Sheet_1.zip › supplemental raw WB pictures/DH82 0636 expression 021522.jpg]

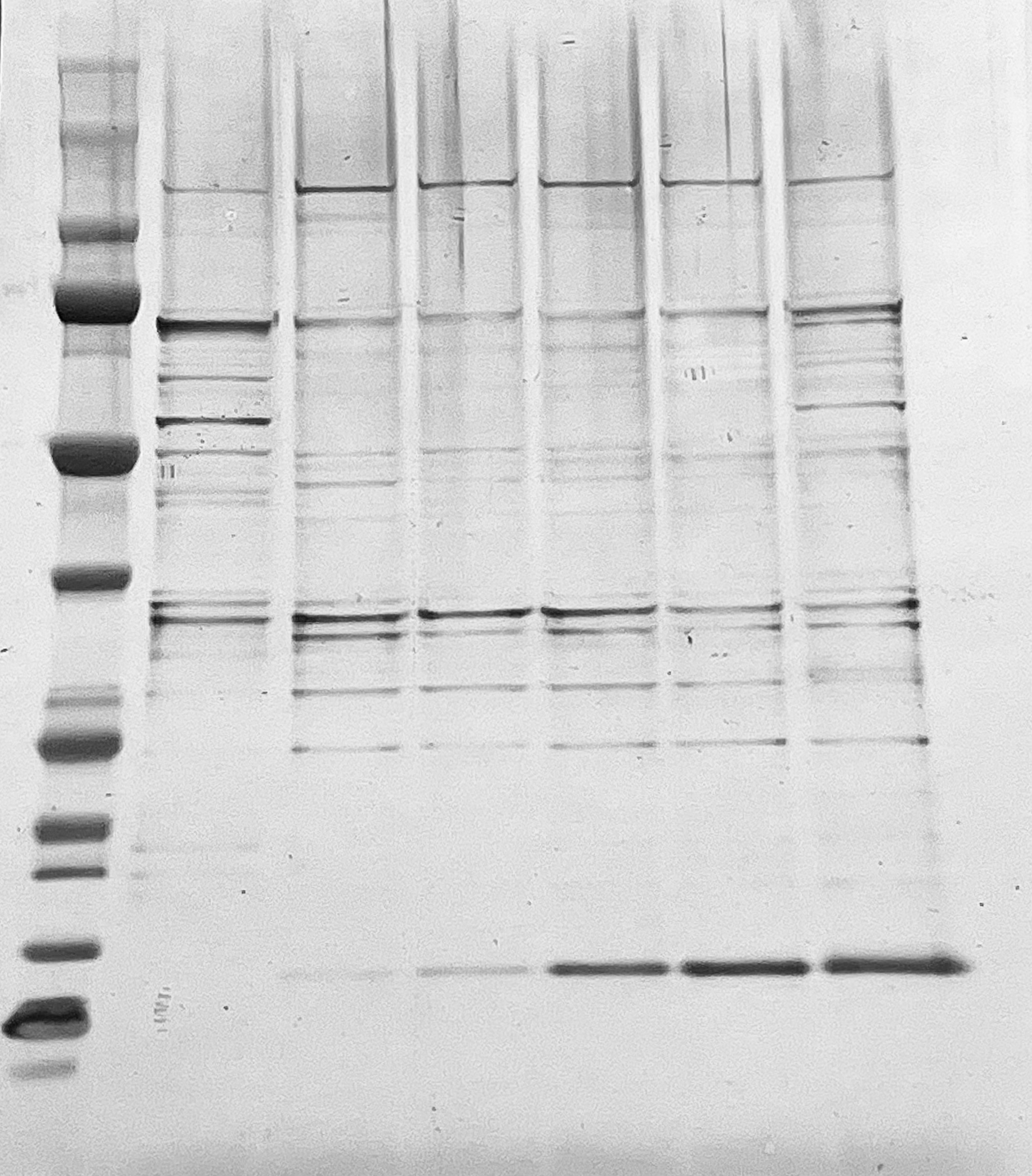

Supplement: Supplementary file 1 [file Data_Sheet_1.zip › supplemental raw WB pictures/DH82 0919 expression 031622.jpg]

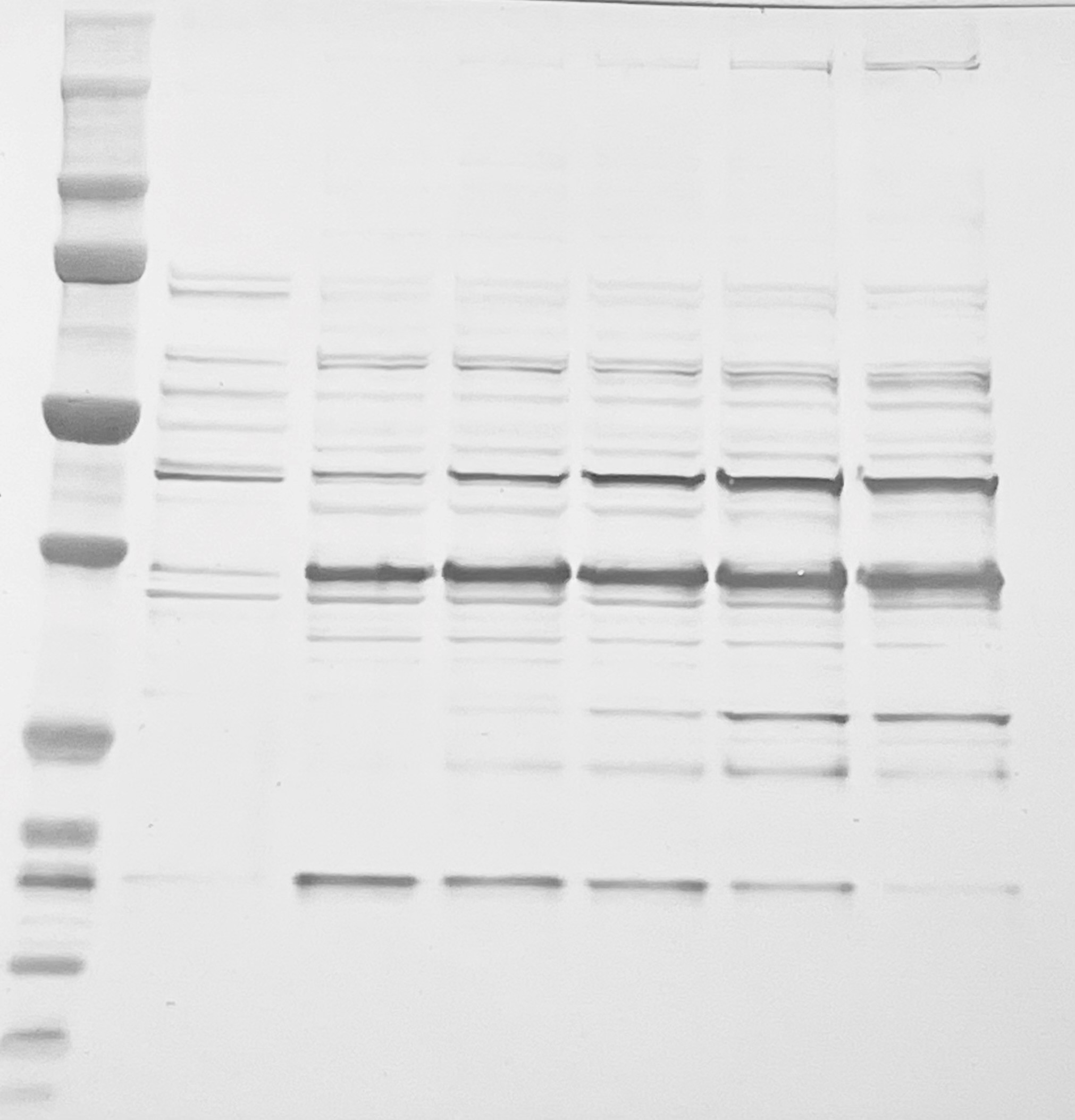

Supplement: Supplementary file 1 [file Data_Sheet_1.zip › supplemental raw WB pictures/DH82 0920 expression 021522.jpg]

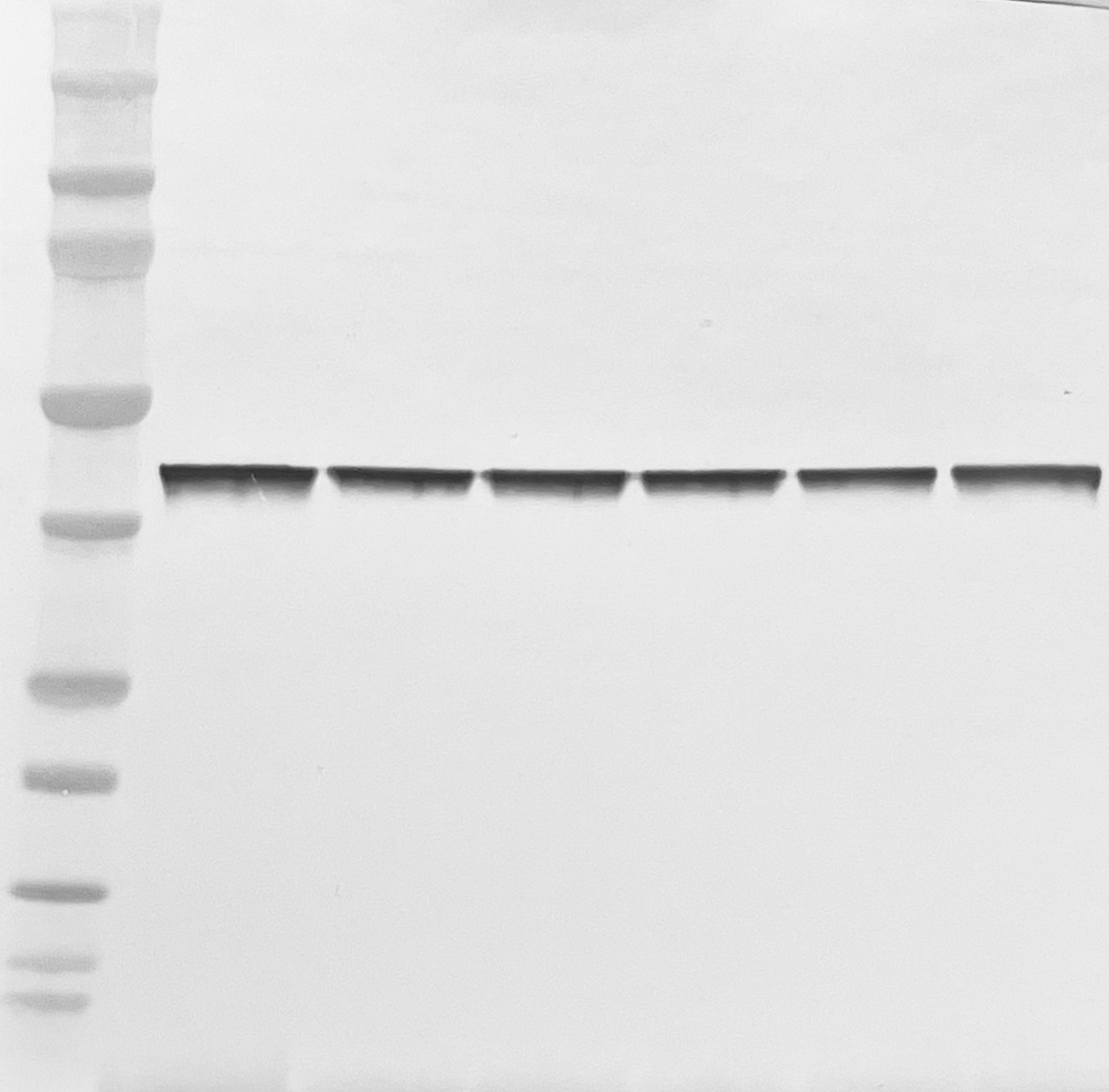

Supplement: Supplementary file 1 [file Data_Sheet_1.zip › supplemental raw WB pictures/DH82 beta actin expression 030322.jpg]

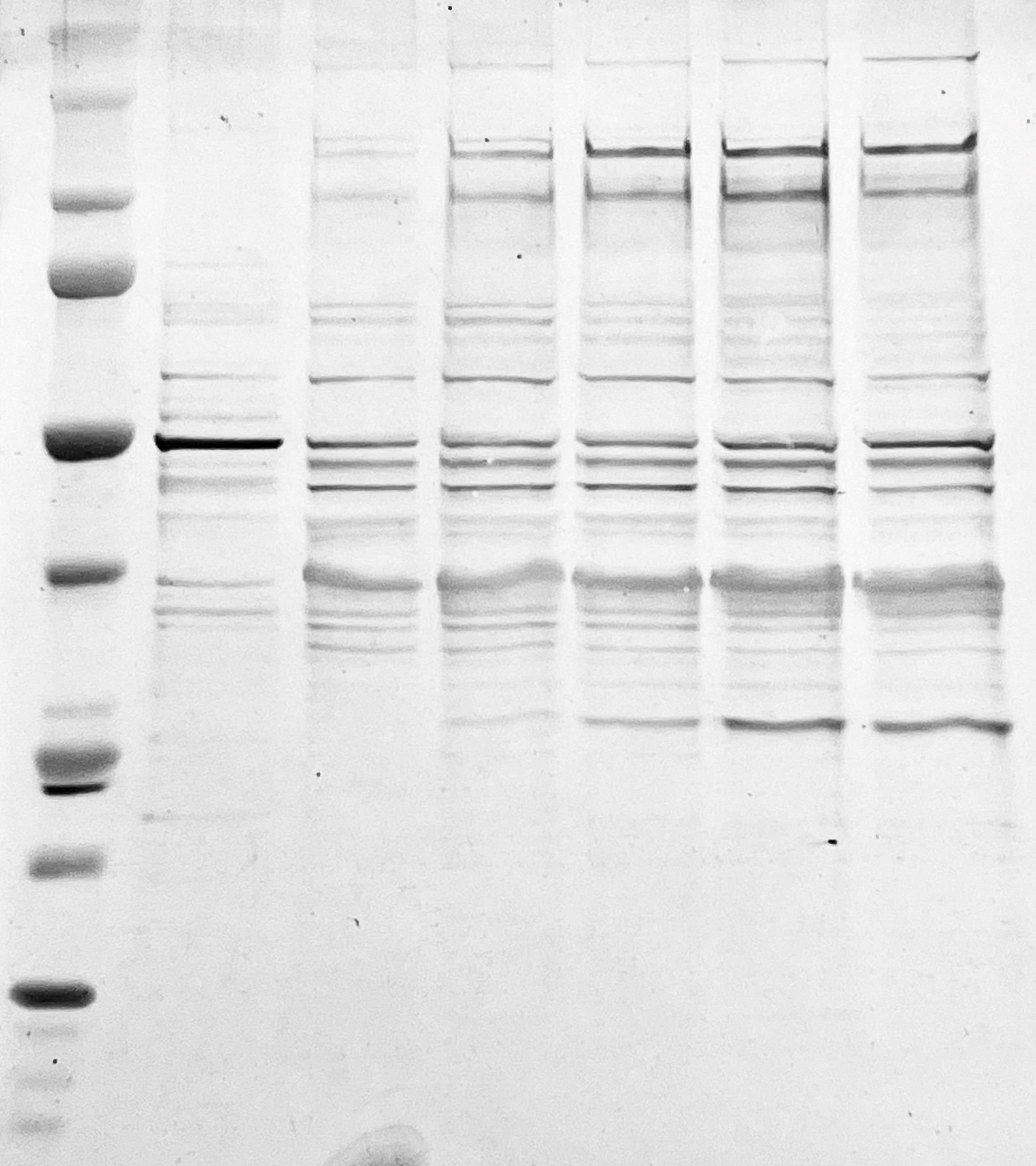

Supplement: Supplementary file 1 [file Data_Sheet_1.zip › supplemental raw WB pictures/DH82 TRP140 expression 021522.jpg]

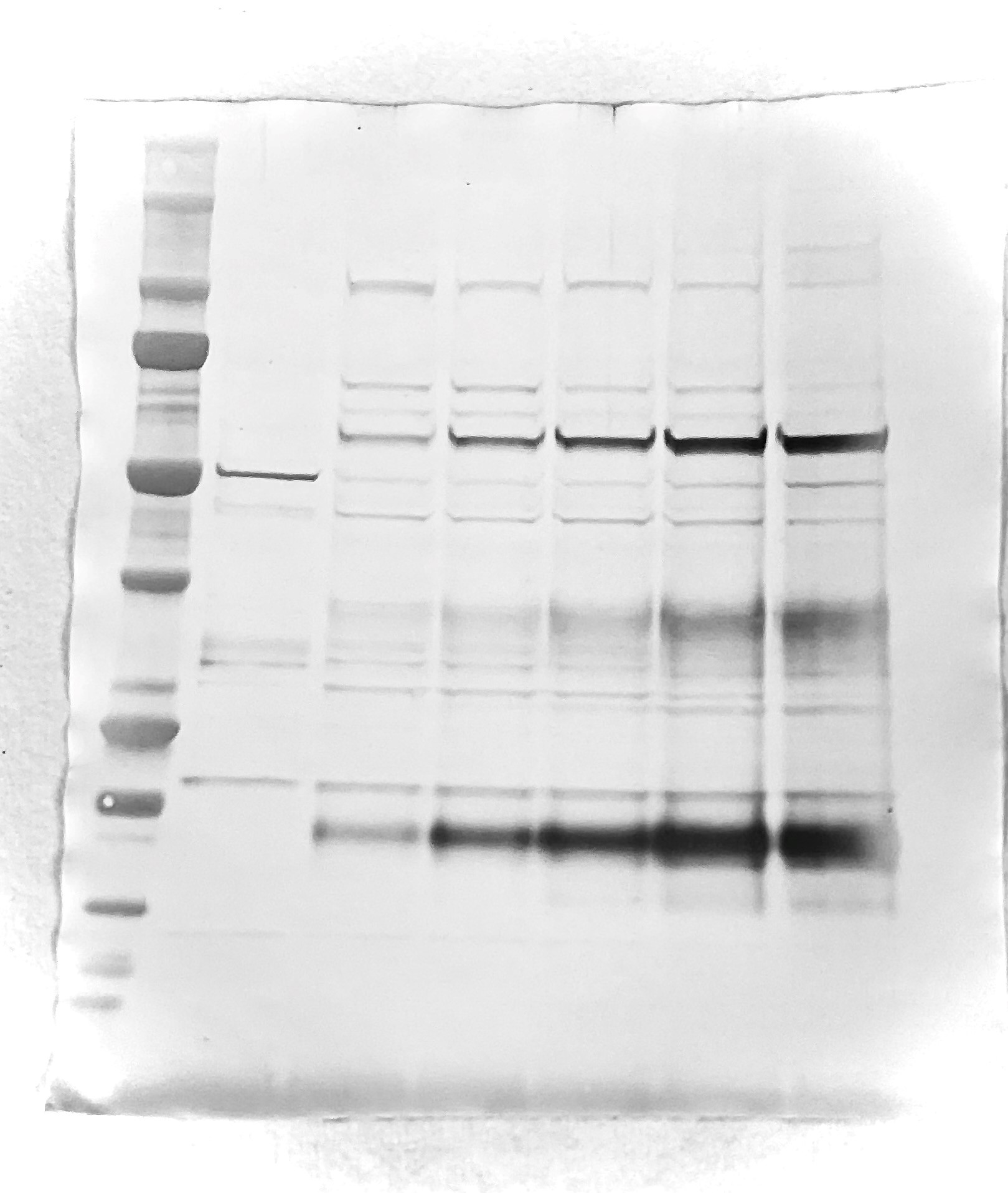

Supplement: Supplementary file 1 [file Data_Sheet_1.zip › supplemental raw WB pictures/DH82 TRP19 expression 2 021022.jpg]

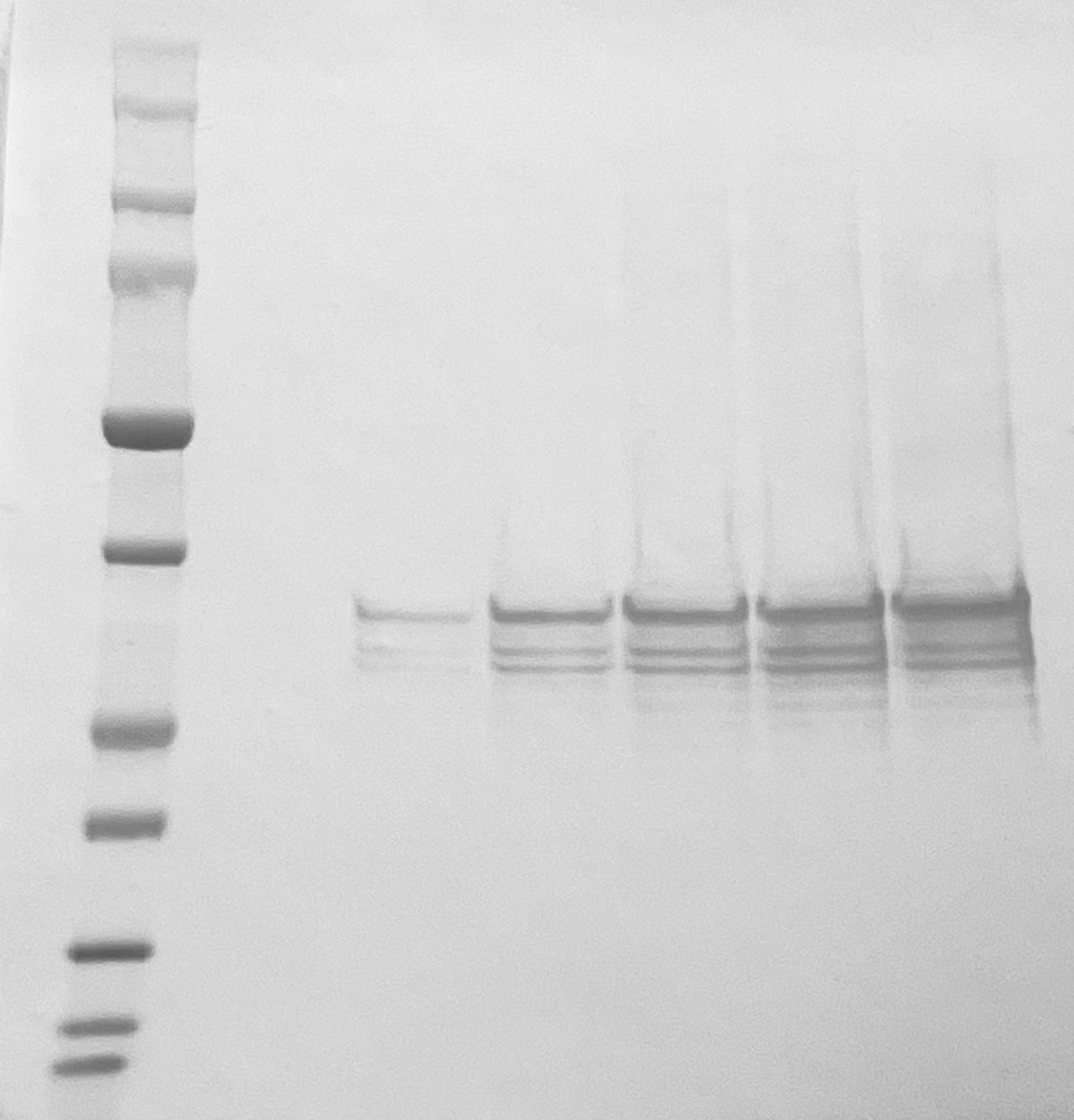

Supplement: Supplementary file 1 [file Data_Sheet_1.zip › supplemental raw WB pictures/DH82 TRP36 expression 1 021822.jpg]

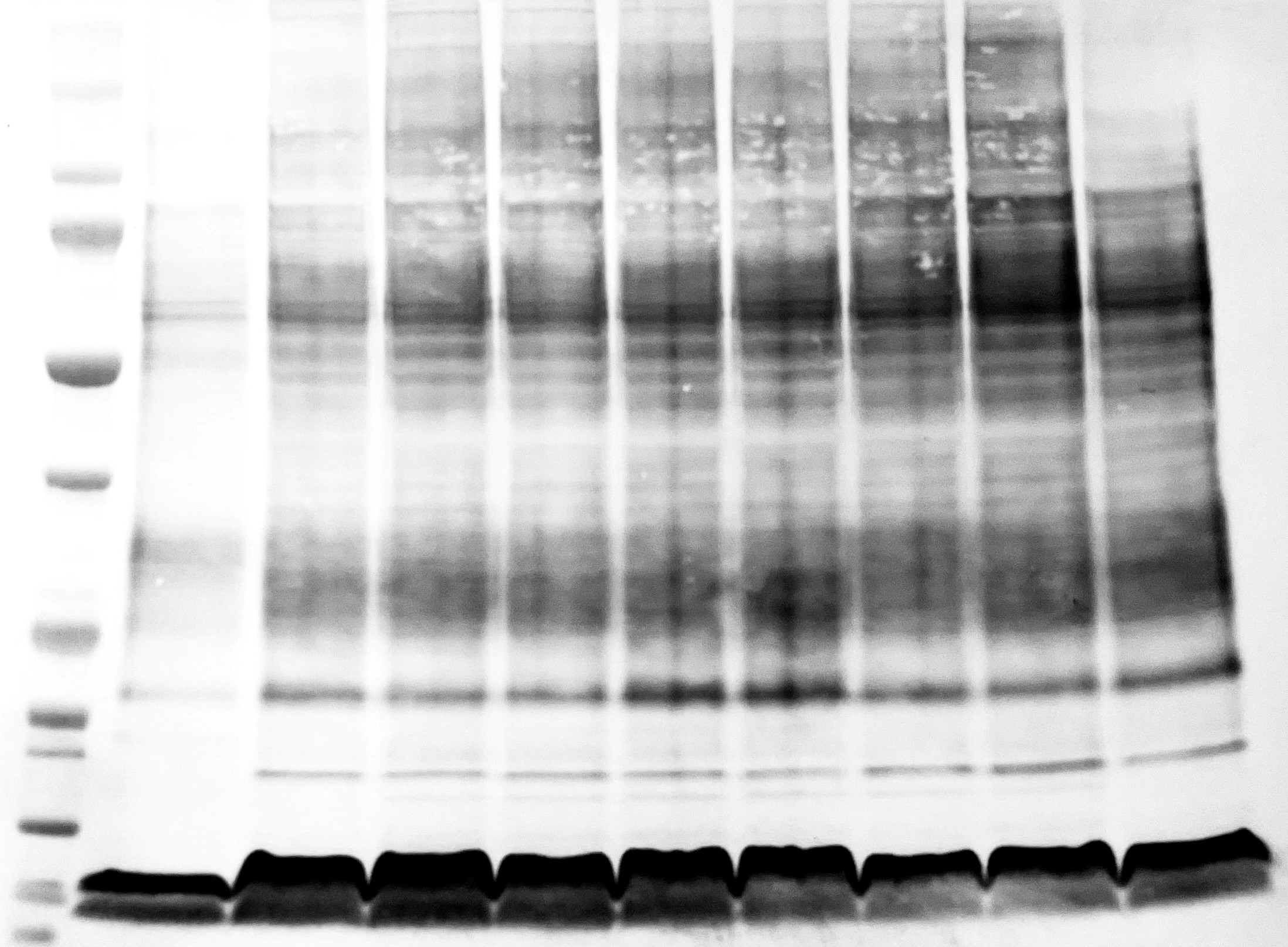

Supplement: Supplementary file 1 [file Data_Sheet_1.zip › supplemental raw WB pictures/ISE6 0073 expression 021122.jpg]

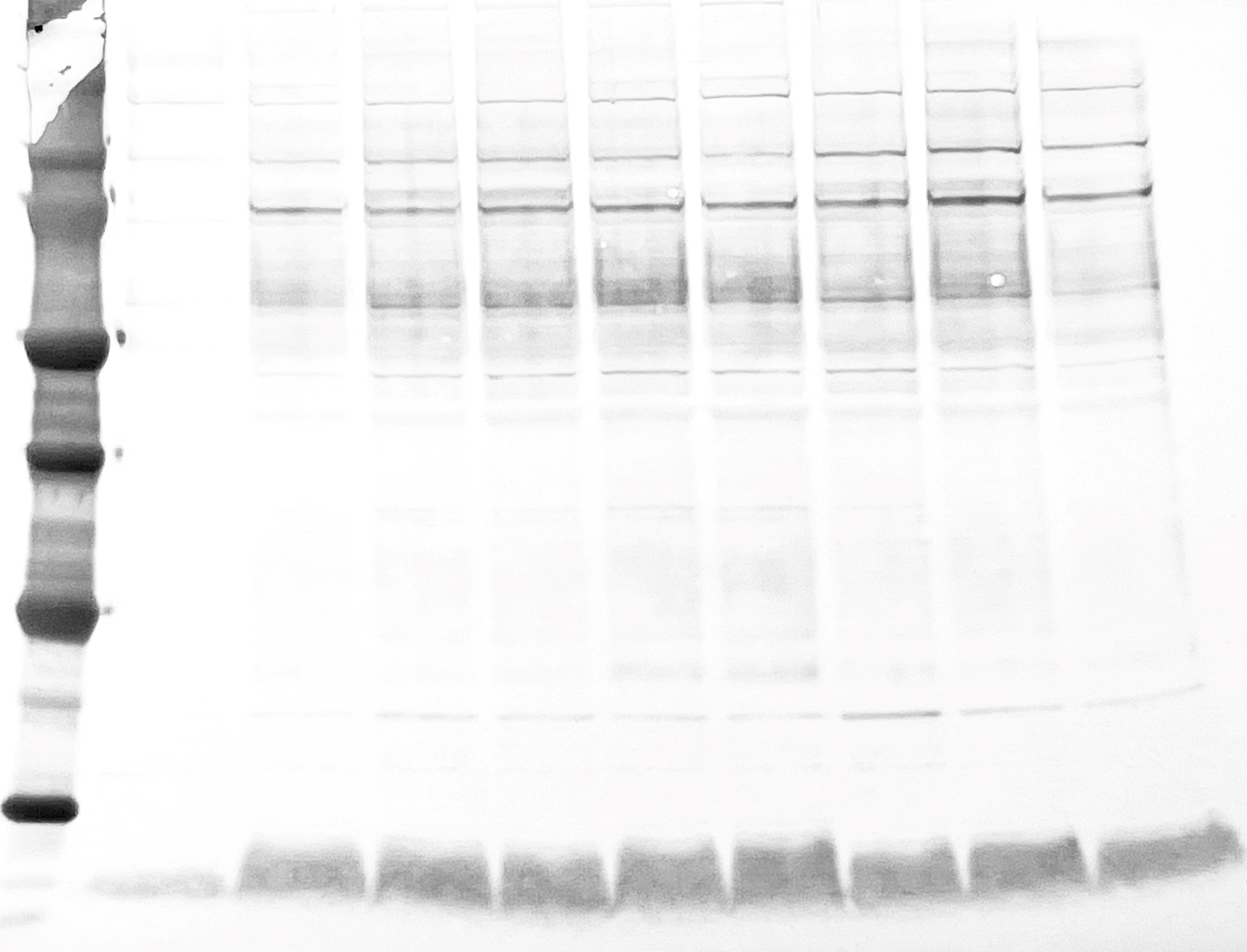

Supplement: Supplementary file 1 [file Data_Sheet_1.zip › supplemental raw WB pictures/ISE6 0126 expression 021622.jpg]

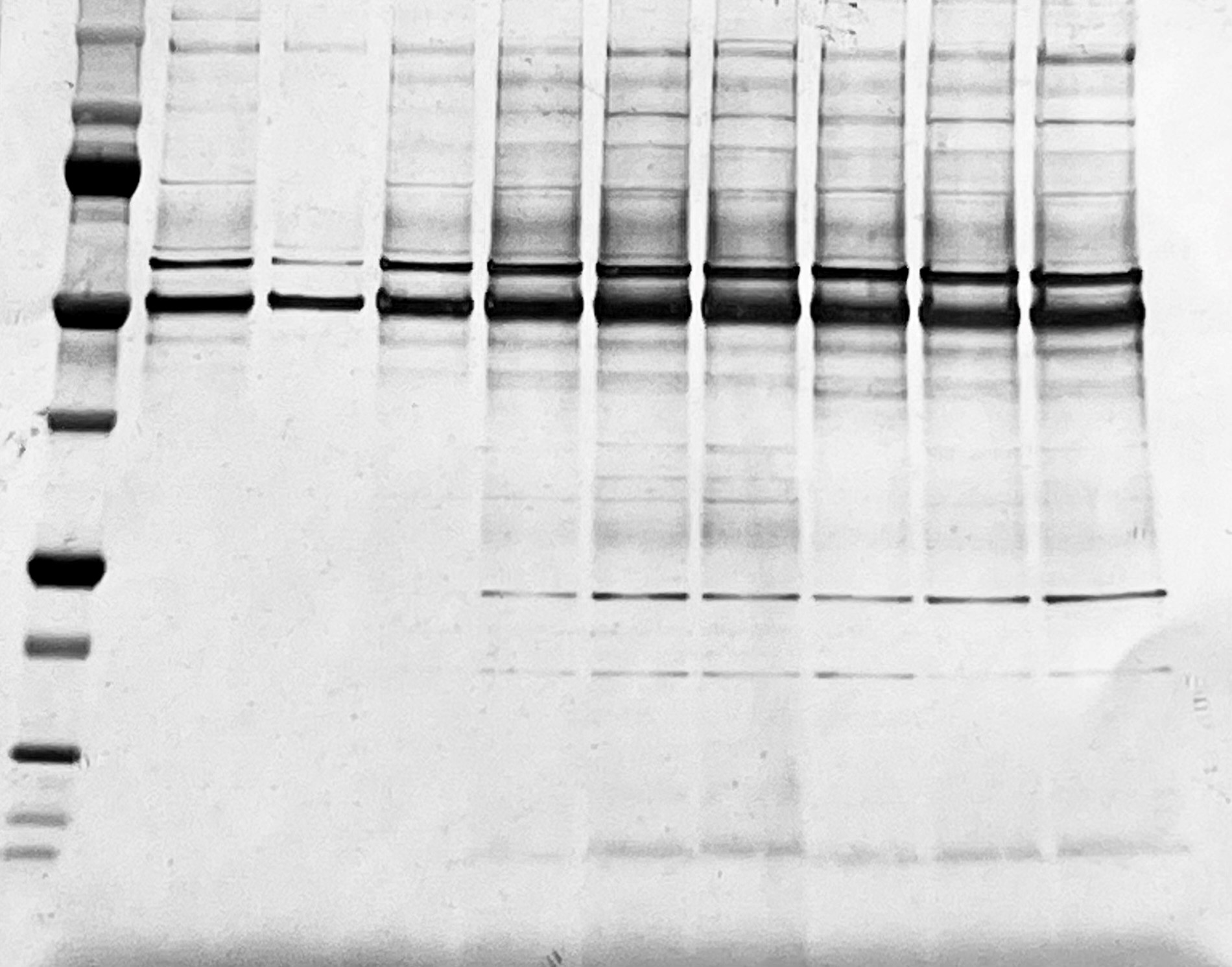

Supplement: Supplementary file 1 [file Data_Sheet_1.zip › supplemental raw WB pictures/ISE6 0636 expression 051722.jpg]

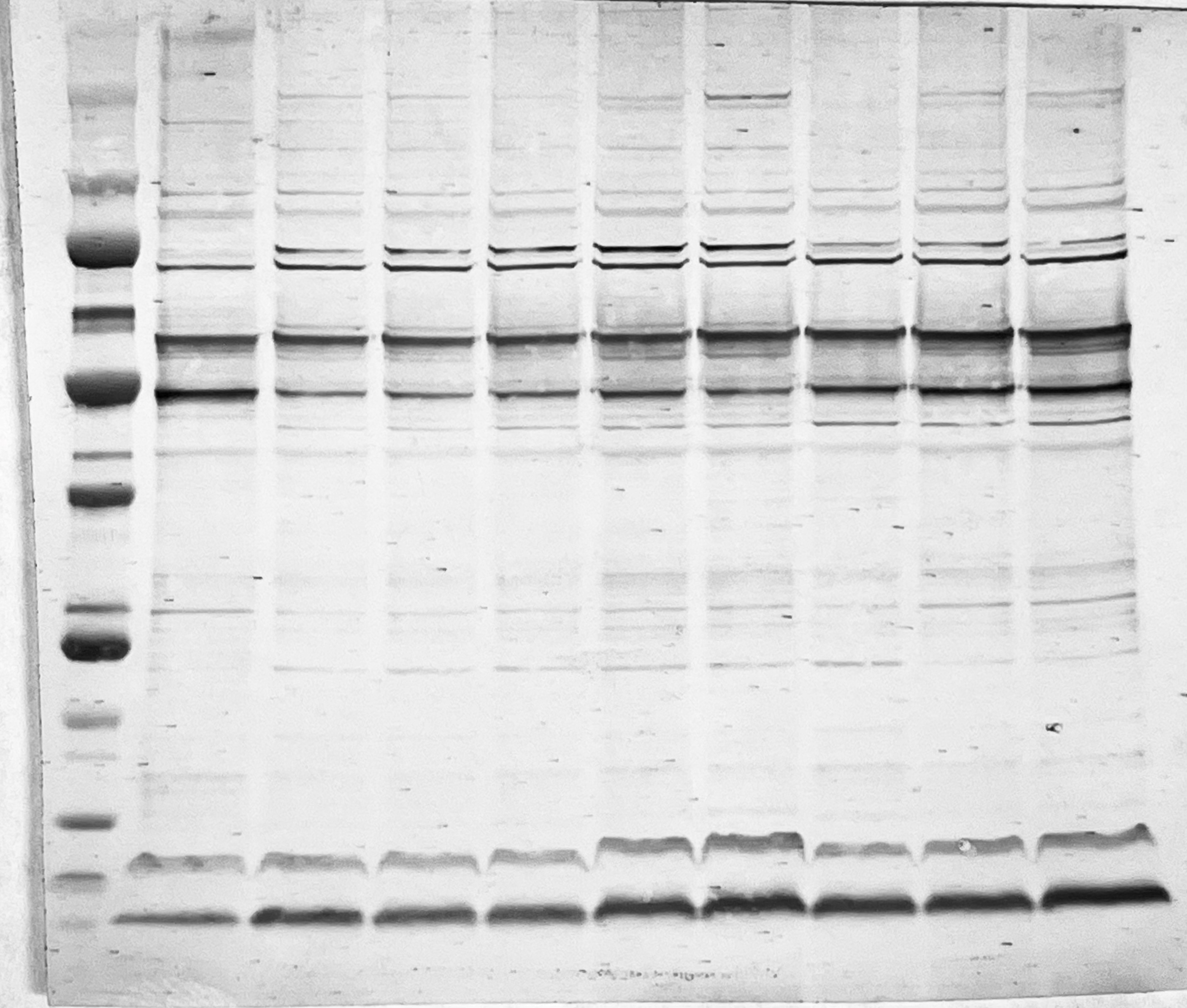

Supplement: Supplementary file 1 [file Data_Sheet_1.zip › supplemental raw WB pictures/ISE6 0919 expression 032522.jpg]

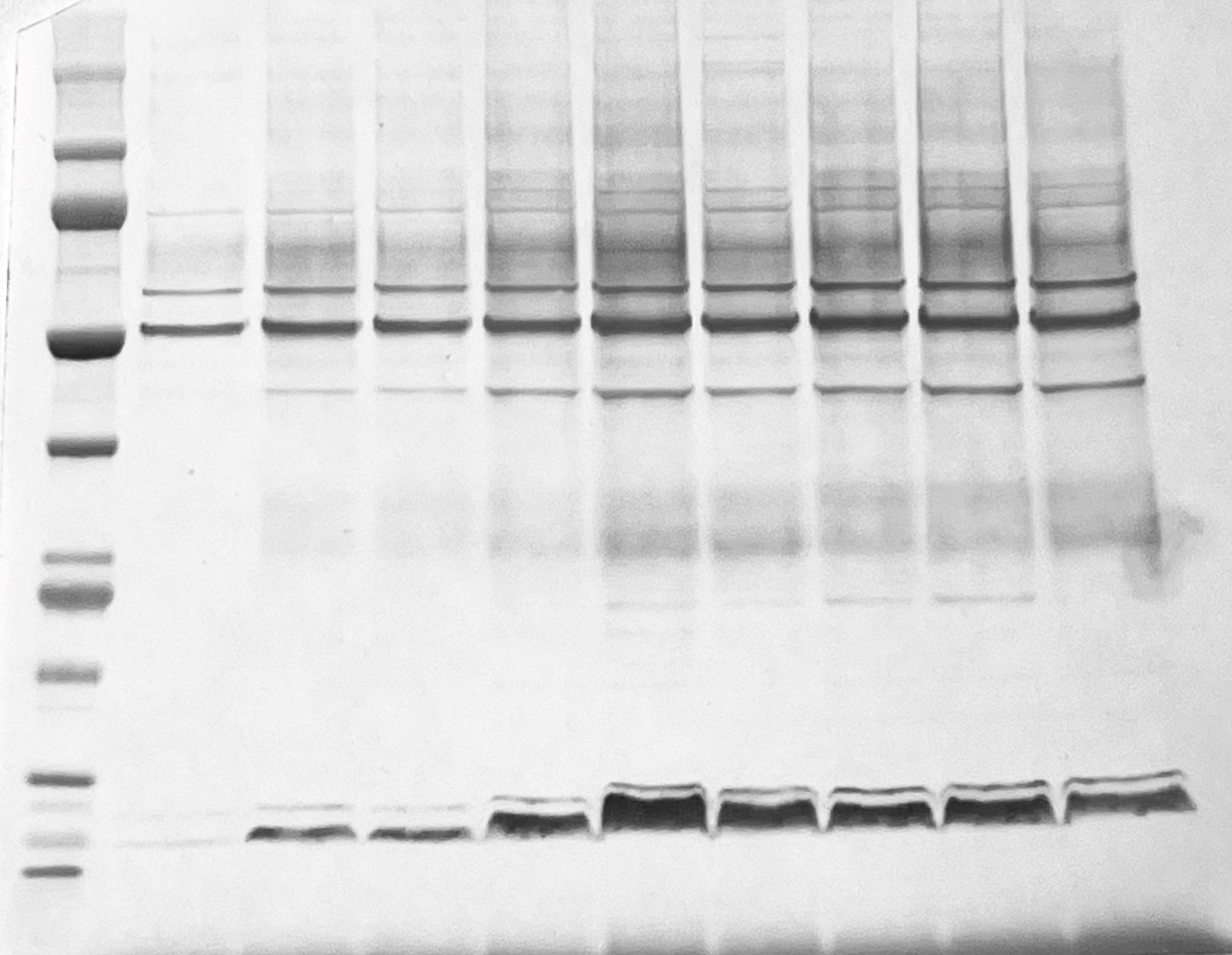

Supplement: Supplementary file 1 [file Data_Sheet_1.zip › supplemental raw WB pictures/ISE6 0920 expression 051222.jpg]

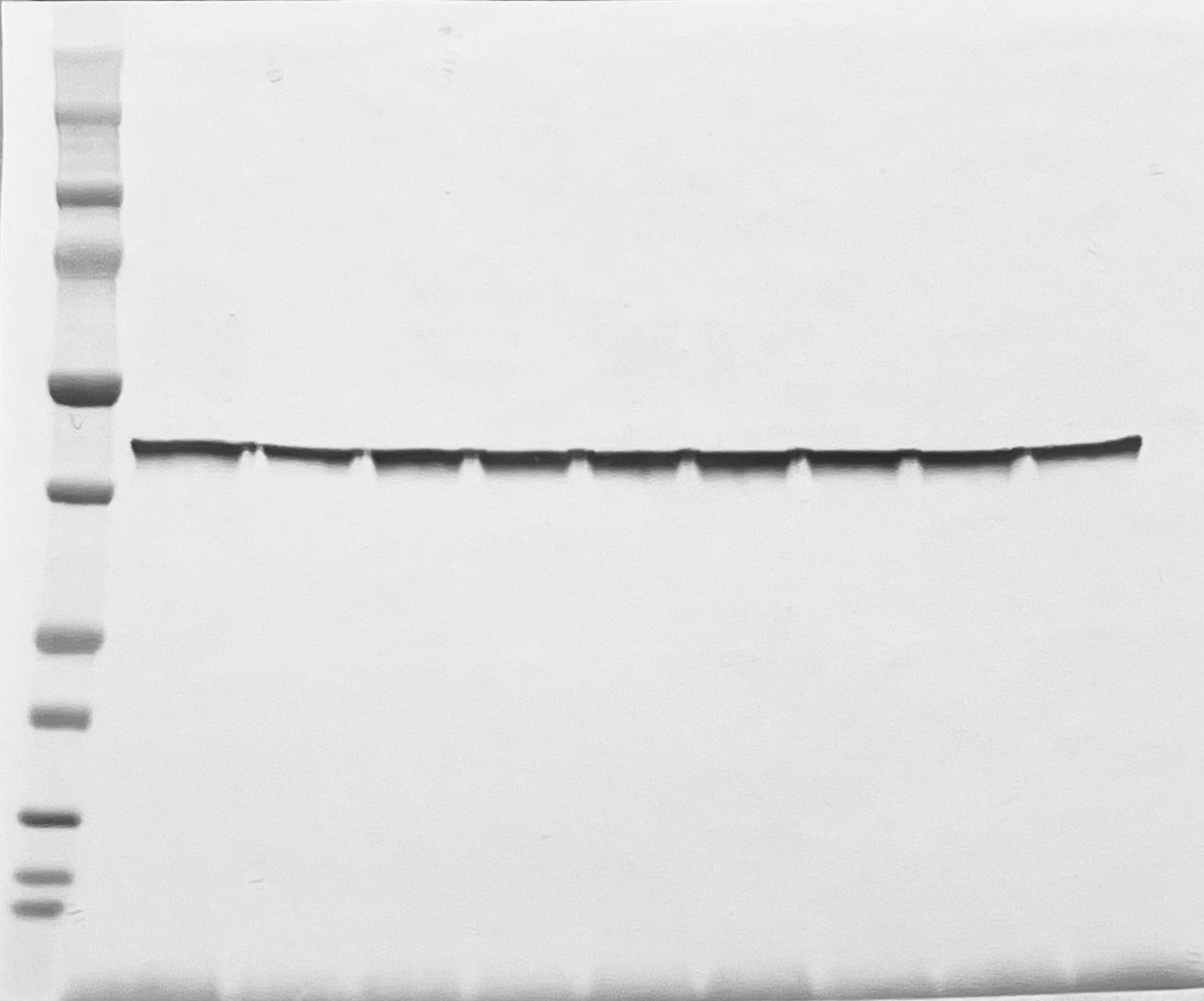

Supplement: Supplementary file 1 [file Data_Sheet_1.zip › supplemental raw WB pictures/ISE6 beta actin expression 030322.jpg]

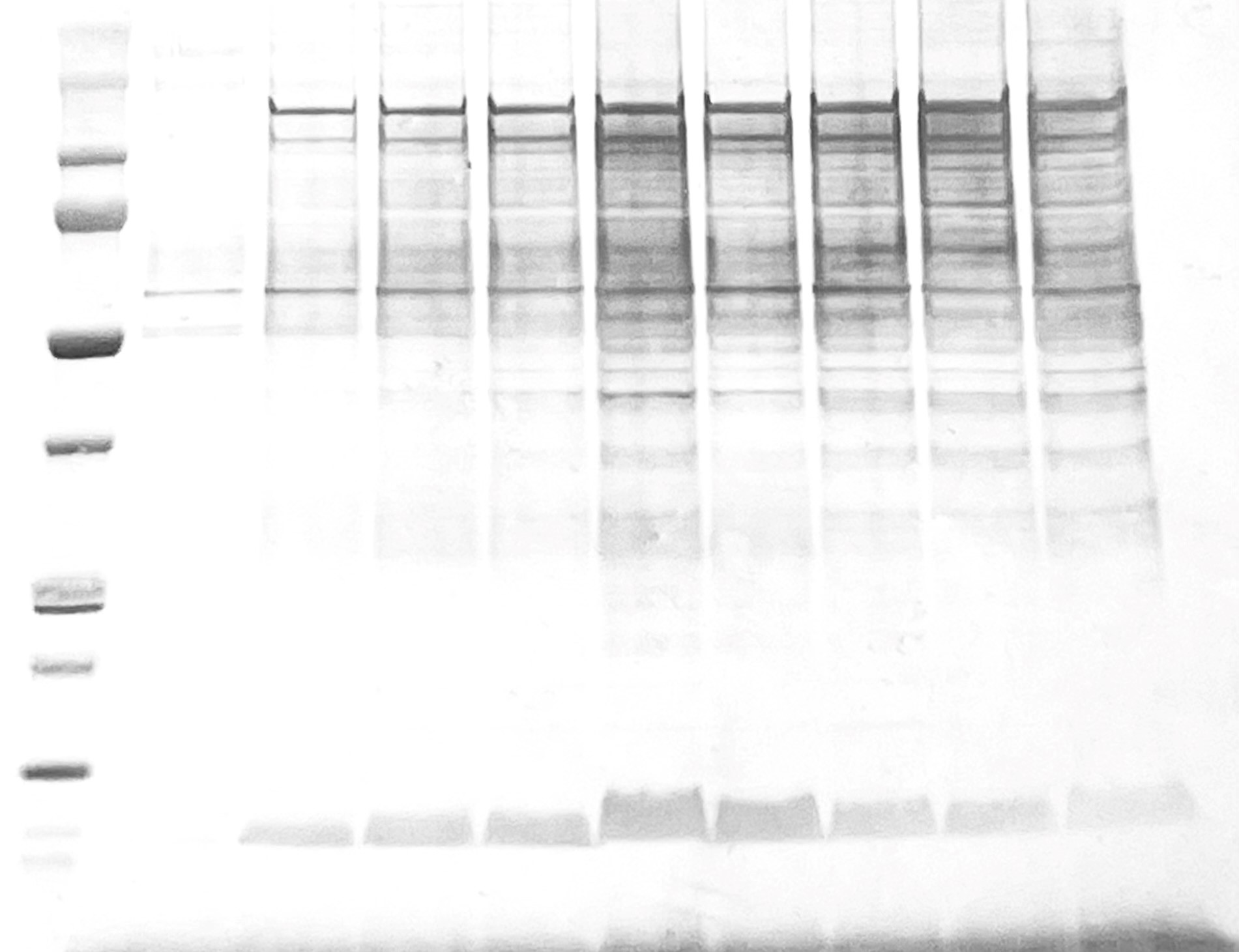

Supplement: Supplementary file 1 [file Data_Sheet_1.zip › supplemental raw WB pictures/ISE6 TRP140 expression 051222.jpg]

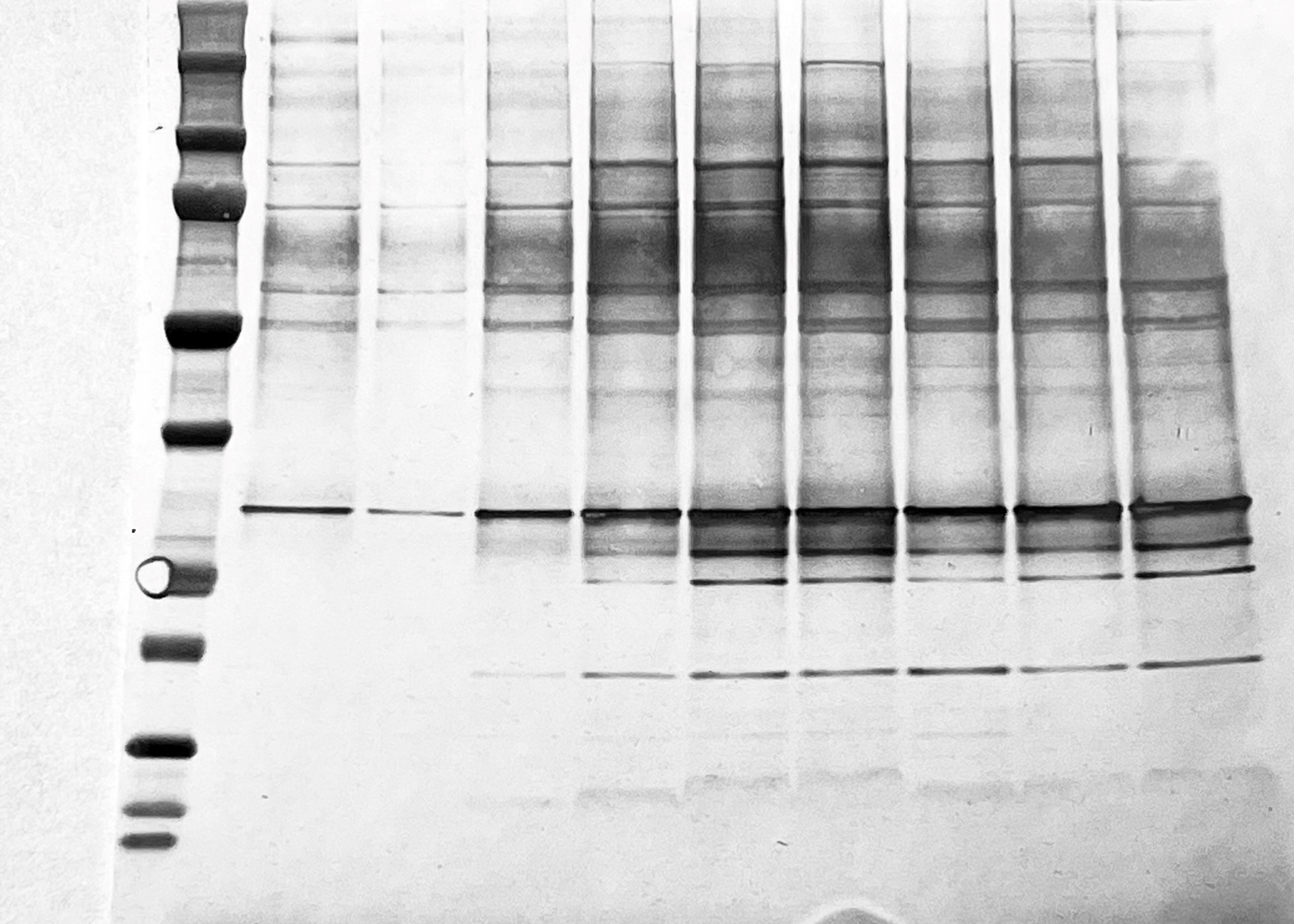

Supplement: Supplementary file 1 [file Data_Sheet_1.zip › supplemental raw WB pictures/ISE6 TRP19 expression 051722.jpg]

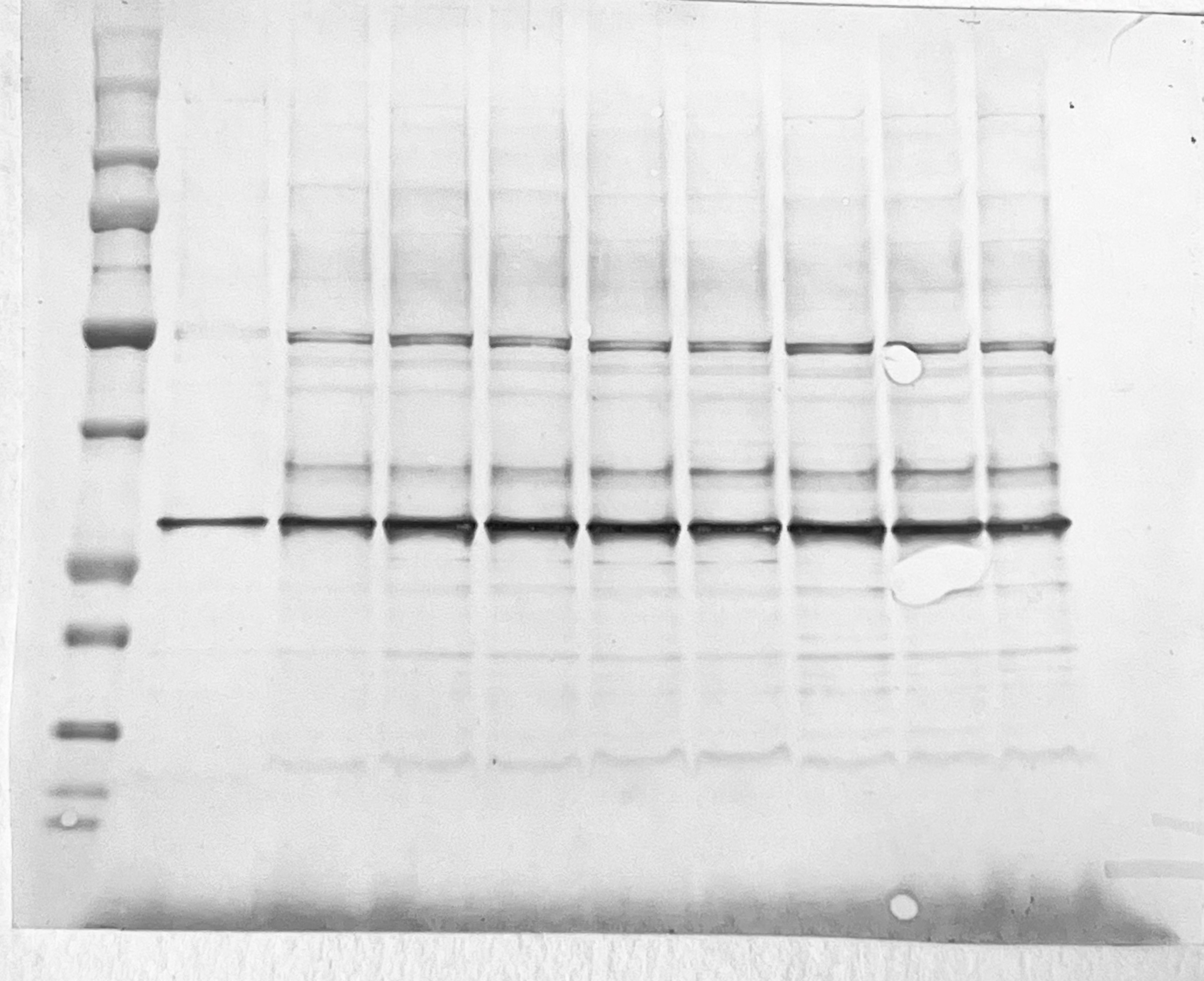

Supplement: Supplementary file 1 [file Data_Sheet_1.zip › supplemental raw WB pictures/ISE6 TRP36 expression 1 021522.jpg]
